# Autonomous helical propagation of active toroids with mechanical action

**DOI:** 10.1038/s41467-019-09099-9

**Published:** 2019-03-06

**Authors:** Bowen Shen, Youliang Zhu, Yongju Kim, Xiaobin Zhou, Haonan Sun, Zhongyuan Lu, Myongsoo Lee

**Affiliations:** 1 State Key Laboratory of Supramolecular Structure and Materials, College of Chemistry, Jilin University, Changchun, 130012 China; 2 State Key Laboratory of Polymer Physics and Chemistry, Changchun Institute of Applied Chemistry, Chinese Academy of Sciences, Changchun, 130022 China

## Abstract

Self-assembly in nature is fundamentally dynamic, existing in out-of-equilibrium state in which the systems have the ability to autonomously respond to environmental changes. However, artificial systems exist in a global minimum state, which are incapable of conducting such complex functions. Here we report that input of thermal energy can trigger fixed, artificial toroids to spontaneously nucleate helical growth. The helical polymerization undergoes reversible and repeatable cycles with subsequent energy input. When the toroids are located inside lipid vesicles, the polymerization-depolymerization cycle is accompanied by reversible elongation of spherical vesicles. Such liberation from a global minimum state will pave the way to create emergent structures with functions as complex as those of living systems.

## Abstract (web summary)

Self-assembly in nature is dynamic and exists in out-of-equilibrium state and thus systems have the ability to autonomously respond to environmental changes. Here the authors report that input of thermal energy can trigger fixed, artificial toroids to spontaneously nucleate helical growth.

## Introduction

Living systems are dynamic, requiring continuous energy input to maintain assembly in the functional state with adaptive and autonomous behavior^1,2^. With energy input, for example, toroidal proteins perform a variety of adaptive functions including DNA replication^3,4^, microtubule severing^5,6^ and nucleation^7^, and protecting genomic materials in filamentous virus^8–10^. Moving from nature to artificial systems, most synthetic assembly exists in a global minimum state^11–19^, thus incapable of exhibiting such complex functions. Applying out-of-equilibrium nature of living systems to artificial assembly would provide an approach for creating the next generation of molecular materials with emergent functions far beyond what static systems can provide^20–22^.

**Fig. 1:**
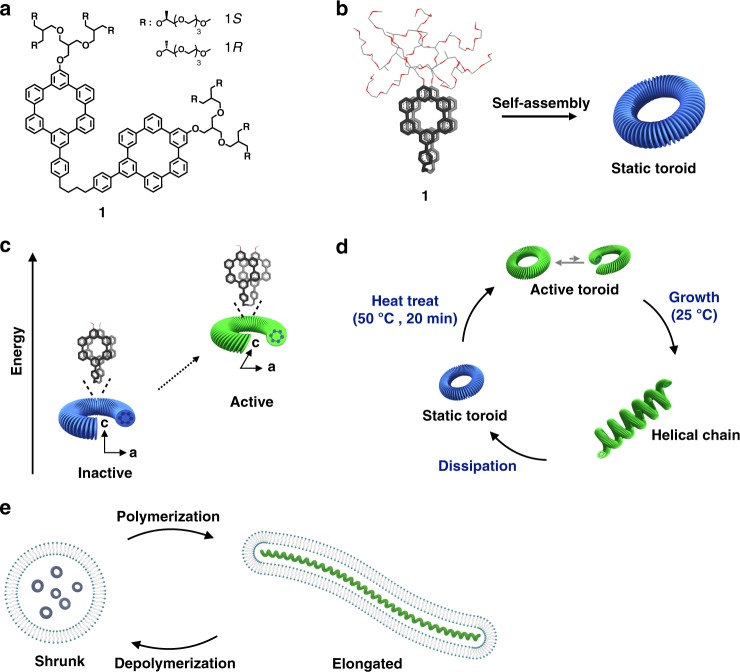
Dissipative helical polymers fueled by heat stress. **a** Molecular structure of aromatic macrocycle 1. **b** Schematic representation of the toroidal assembly of 1 with an eclipsed conformation. **c** Activation of the toroids with a tilting transition by applying heat stress as an energy source. **d** Schematic representation of the helical polymerization and depolymerization cycle of the toroids upon heat treatment. **e** Schematic representation of elongation/contraction motion of the lipid vesicle encapsulating toroids driven by polymerization and depolymerization cycle

Here we report that heat stress as an energy source can trigger synthetic toroidal objects to undergo autonomous helical propagation and random collapse, accompanied by work generation. The polymerization and depolymerization cycles are repeatable with subsequent energy input without sacrificing energy efficiency. Importantly, the polymerization–depolymerization cycle generates elongation/contraction motion of lipid vesicles when encapsulate the toroids inside (Fig. 1).

## Results

### Molecular design and self-assembly

A switchable event in a subunit level of proteins, such as a conformational change particularly in response to stressful circumstances, can trigger its higher-level assembly to exhibit emergent functions^23,24^. The design of molecules capable of a conformational switch involves a dimeric disk-shaped aromatic core incorporating hydrophilic oligoether dendrons which adopts a folded conformation in hydrophilic environments. The folded aromatic dimer is able to undergo a transition between eclipsed and slipped conformations driven by thermal dehydration of the oligoether chains^25^. This conformational switch of a molecular component can trigger its self-assembled structures to be active, enabling them to evolve autonomously into an emergent structure. To realize this goal, we incorporated bulky dendritic chains into both ends of the aromatic dimer which is expected to interrupt rapid switching back into a favorable eclipsed conformation due to strong steric crowding of the two bulky dendrimers in the folded state (Fig. 1a).

**Fig. 2:**
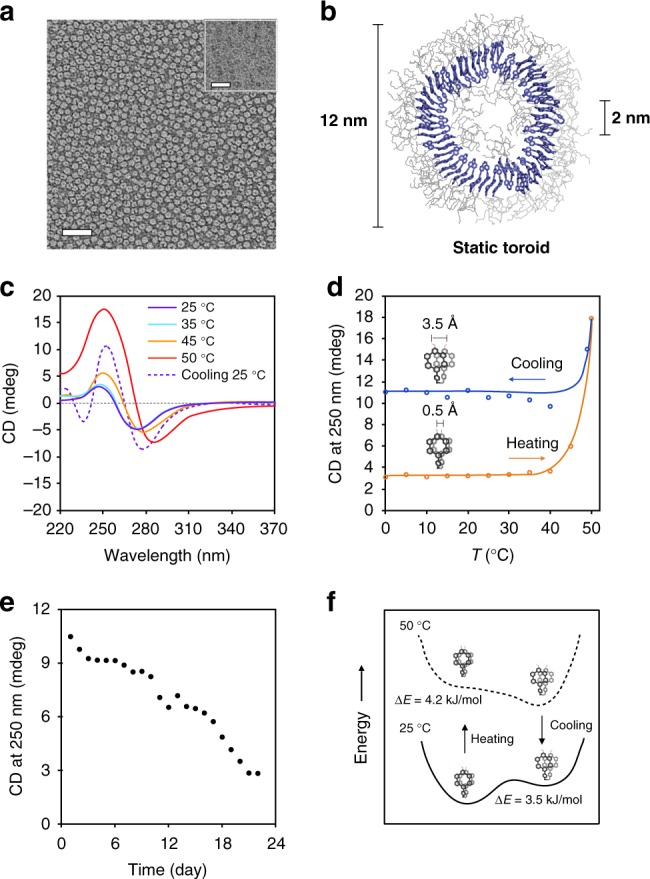
Characterization and activation of toroids by heat treatment. **a** Negatively-stained TEM image (scale bar, 50 nm; inset, cryo-TEM image, scale bar, 25 nm) of toroids formed by 1*S* (30 μM) in aqueous (10 vol% THF) solution. **b** Calculated static toroidal structure from MD simulations. **c** Temperature-dependent circular dichroism (CD) spectra of 1*S* (30 μM) in aqueous (10 vol% THF) solution. **d** Thermal hysteresis of 1*S*, temperature-dependent CD intensity changes at 250 nm (orange, heating curve; blue, cooling curve). **e** Relaxation of 1*S*, time-dependent CD intensities at 250 nm after heating to 50 °C and then cooling to room temperature. **f** Energy diagrams of eclipsed and slipped conformations at different temperatures

In an aqueous solution including 10% (v/v) of THF of dimeric molecule 1 (Supplementary Figures 1 and 2) adopts a folded conformation, generating a wedge-shaped molecular geometry with highly crowded dendritic chains at the exterior (Supplementary Figures 3–6). Consequently, the molecular wedges are able to self-assemble into highly-curved structures^26^. Indeed, transmission electron microscopy (TEM) of 1 showed the formation of highly uniform donut-like objects with an external diameter of 12 nm (Fig. 2a and Supplementary Figure 7). The well-dispersed toroidal objects in solution showed to be highly stable, remained unchanged over a period of 2 years without any deformation or any further aggregation, consistent with the all-atom molecular dynamics simulation result (Fig. 2b and Supplementary Figure 8, Movie 1).

### Activation of toroids by heat treatment

Upon heating, the thermal dehydration at about 50 °C of the oligoether chains located at the toroidal exterior (Supplementary Figures 9 and 10) leads the eclipsed packing of the dimeric macrocycle to be slipped against one another (Supplementary Figures 11 and 12) due to an increased cross-sectional area of the globules and strengthened hydrophobic interactions^25,27^. This is reflected in a sharp increase in the intensity of a Cotton effect and red shifted UV absorption of 1*S* at 50 °C (Fig. 2c and Supplementary Figure 13). The notable increase in the CD intensity indicates that the slipping undergoes in a preferred direction caused by the chiral transfer from the asymmetric centers in the dendritic chains. This is also illustrated by a mirror-image relationship of the CD signals with an opposite enantiomer, 1*R* (Supplementary Figure 14).

The slipped conformation of the dimer has a high kinetic stability, characterized by a large hysteresis (Fig. 2d and Supplementary Figure 15). Upon cooling from 50 °C, the CD intensity at 250 nm of 1*S* decreases up to 40 °C and then remains unchanged. At room temperature, the increased CD intensity slowly relaxes to recover its original intensity over a period of 20 days (Fig. 2e and Supplementary Figure 16). The hysteresis effect in CD intensity is due to the slipped packings being kinetically trapped to reside at a local minimum in the energy landscape (Fig. 2f and Supplementary Figure 17). The kinetic trapping arises from the minimization of π–π repulsion in the slipped packings of the dimeric stack and severe steric crowding of the dimeric dendrons interrupting switching back into eclipsed packing^28^.

Notably, the toroidal structures remained unchanged even after heat treatment. When exposed to heat at 50 °C for 20 min and then cooled to room temperature to stand for 10 min, TEM and AFM revealed that the toroidal structures of 1 retain without compromising their intact structure (Supplementary Figure 18), indicating that heat treatment leads the toroid to undergo a tilting transition of the constituent aromatic discs. In the solution before heat treatment, the dimeric macrocycle discs with eclipsed packings are approximately perpendicular to the plane of the toroid to maximize aromatic interactions. Upon heating, however, the dehydrated oligoether dendrons would make the eclipsed stackings of the discs to be unstable due to steric hindrance between the globular-shaped dendrons with a greater cross-section^25^. To relieve the steric hindrance at the interface, the disc planes would be tilted through the slipping with respect to each other to allow a larger interfacial area (Fig. 1c), thus lowering total free energy^29^.

**Fig. 3:**
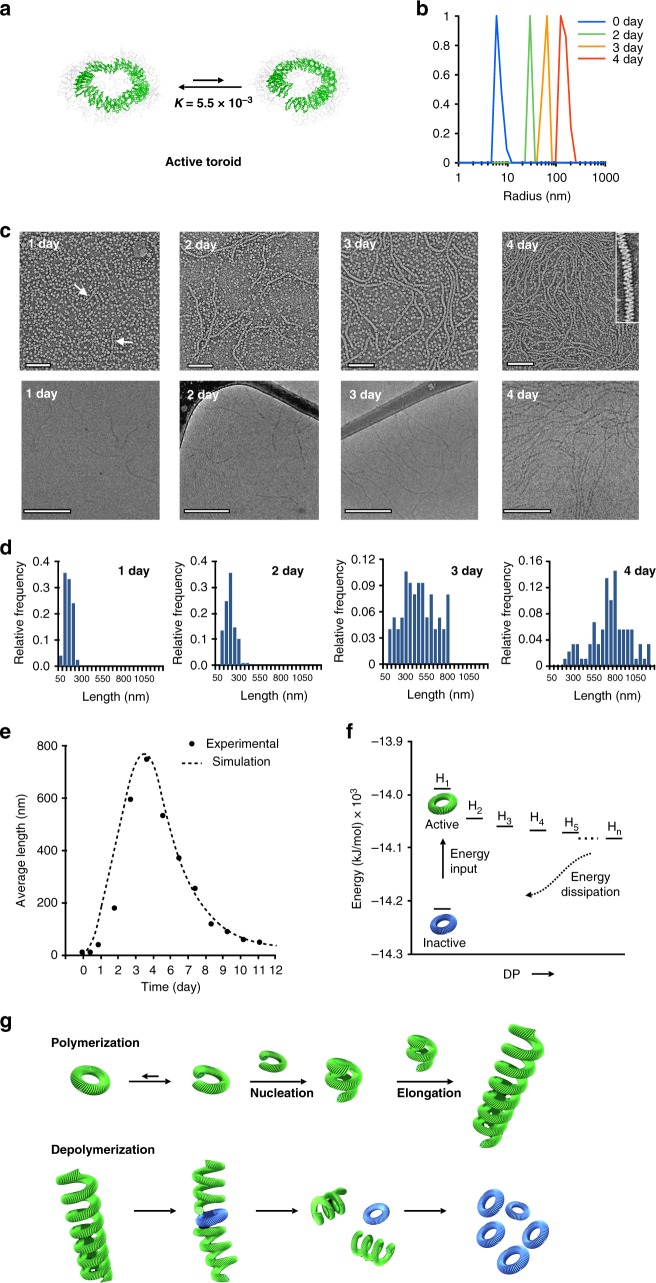
Autonomous helical polymerization and depolymerization. **a** MD simulations of active toroids exhibiting dynamic equilibrium between closed form and spirally open form. **b** DLS profiles of 1*S* (30 μM) in aqueous (10 vol% THF) solution after heat treatment at 50 °C for 20 min. **c** Time-dependent negatively-stained TEM (top, scale bar, 100 nm) and cryo-TEM (bottom, scale bar, 200 nm) images of 1*S* (30 μM) in aqueous (10 vol% THF) solution after heat treatment. The arrows in the one-day image indicate the formation of short helices. Inset of the 4-days image is magnified image exhibiting a right-handed helical structure. **d** Time-dependent histograms of the chain length distributions, counted from the cryo-TEM images (*n* = 100). **e** Time-dependent average chain lengths, counted from cryo-TEM images (*n* = 100), and fitted curve from kinetic Monte Carlo simulations (dotted curve). **f** Energy variation representing polymerization and depolymerization of toroids upon energy input. **g** Schematic representation of polymerization of active toroids (green) and depolymerization into static toroids (blue)

To gain insight into the conformational dynamics of the toroid based on a tilted packing arrangement, we performed molecular dynamics simulations using a coarse-grained model (Supplementary Figure 19). The simulations showed that a dynamic equilibrium could be established between closed ring and open spiral conformations (Fig. 3a and Supplementary Movie 2). With the tilted packings based on a slipped arrangement of the dimeric discs, the closed toroid became dynamically open, characterized by a spiral conformation with two open ends. The equilibrium constant of an open form over a closed toroid was calculated to be 5.5 × 10^−3^. Thus, heat-treatment as an energy source drives the toroids to reside in kinetically trapped state, far from their thermodynamic equilibrium.

### Spontaneous helical polymerization and depolymerization

As a result of the exposure of the hydrophobic cross sections of the open form to hydrophilic environment, the toroidal objects have sufficient activity to autonomously nucleate helical structuring through end-to-end connection. Indeed, DLS experiments with the solutions of 1 at room temperature after heat treatment at 50 °C for 20 min revealed that heat treatment triggers the size of aggregates to remarkably increase in hydrodynamic diameter from 12 nm to ~250 nm over a period of 4 days (Fig. 3b). To further identify the structural evolution of the toroids, TEM experiments were performed with the solution of 1*S* (Fig. 3c, d and Supplementary Figure 20). When heat treated at 50 °C for 20 min and then cooled to 25 °C, the image revealed that the toroidal structures are maintained without any structural changes. After 1 day standing, however, we found the formation of short helical chains, suggesting that the closed toroid can be spirally open to nucleate helical growth (Fig. 3c, the arrows in the first day image). After a short lag period, indeed, the chain length increases rapidly and then reach to maximum lengths of nearly a micrometer in 4 days (Fig. 3d, e), demonstrating that, once exposed to heat, the toroidal objects are able to hierarchically polymerize to form long helical chains. The polymerization reaction is further supported by a magnified image which revealed the formation of helically-folded polymer chains (Fig. 3c, inset of 4th day image and Supplementary Figure 21), which excludes toroidal stacking.

Close examinations of the images revealed that the helical chains are right-handed (Supplementary Figures 22 and 23). The diameter (12 nm) of the helix is in good agreement with that of the individual toroids, indicating that the helix retains the toroidal curvature. This observation provides further evidence that the helical growth is initiated by the open spiral conformation of a heat-treated toroid. As a result, the toroids with open spiral forms are stabilized by helical growth due to neighbor-turn interactions along the helix^30^. As opposed to 1*S*, the enantiomer, 1*R*, generates left-handed helical chains (Supplementary Figure 24), indicating that the chirality of the oligoether dendron transfers to the helical chain. To support that the open spiral form arises from tilting in one direction, we performed control experiments with the toroidal objects based on a racemic mixture, 1*S*/1*R* that are incapable of tilting in one direction (Supplementary Figure 25). In great contrast to the chiral toroids, indeed, the racemic toroids are unable to polymerize at an identical experimental condition to the solution of 1*S*. This result demonstrates that the disc tilt in one direction is essential for the spiral opening of toroids and helical polymerization.

Notably, from 5 days standing of the heat-treated solution, the helical polymers autonomously shorten in length with irregular chain scission and then collapse to restore the intact toroidal objects after additional 7 days (Fig. 3e, Supplementary Figure 20). After a certain period of time, the polymer chains based on kinetically-trapped, slipped packing of the dimers would be relaxed into eclipsed packing, which drives equilibrium toward static toroids.

To gain insight into the polymerization mechanism, we performed kinetic Monte Carlo simulations based on three independent dynamic processes (Fig. 3e, curve fit and Supplementary Figure 26). The best fit to the observed data indicates that the helical polymerization undergoes with a cooperative nucleation-elongation process^31,32^. The first step of polymerization, i.e., the formation of toroidal dimers, takes place slowly. Once dimer forms, the helical chains elongate rapidly by connecting with spirally-open toroids or helical chains through cooperative interactions (Fig. 3g). At the initial stage, polymerization rate is larger than depolymerization rate, because the toroids are mostly active. With time lapse, the polymerization rate decreases gradually due to relaxation into inactive toroid, as manifested by CD decay (Fig. 2e). When the rate of depolymerization becomes higher than that of polymerization, the length of helical chains begins to decrease and, eventually, collapse to restore its constituent toroids. The results show that the polymerization processes exhibit complex dynamic behavior, where growth and collapse coexist, similar to the dynamic behavior of microtubules^33^.

As mentioned above, the polymerization undergoes through connection between spirally-open toroids and/or helical chains, in which the rate is concentration dependent (Supplementary Figure 27). At higher concentrations, the polymerization rate showed to be higher because of the higher probability of the connections between spirals and/or helical chains. However, the depolymerization showed to be concentration independent, consistent with the observed first order CD decay.

The simulations based on a coarse-grained model revealed that discrete toroidal objects reside at the global minimum in the energy landscape (Fig. 3f and Supplementary Figure 28). Heat treatment of the toroids as an energy source, however, results in dramatic energy increase. To release free energy, the activated toroids nucleate spontaneous helical growth to form hierarchical structures with helical order which become more stable. When the thermal energy is consumed over time, the helical polymers collapse spontaneously into their toroidal subunits in the global minimum of the energy landscape. This result together with a polymerization–depolymerization cycle followed by CD decay (Fig. 2e) indicates that energy input triggers helical polymerization of stable toroids in a global minimum state via switching into spirally-open toroids out-of-equilibrium. The input energy is consumed to keep the proportion of slipped macrocycles high above the critical concentration for generating spirally-open toroids or maintaining helical chains, so that the subunits associate with open toroids and/or helical chain ends and the helical chains grow. After the spirally-open subunits are polymerized into a helical polymer over 4 days, the subunits begin to relax into their equilibrium state with releasing energy. Once equilibrium toroids forms in the helical chains, the polymers begin to autonomously collapse (Fig. 3g). Thus, the energy stored in the helical polymer is dissipated as the helical chains are depolymerized to recover stable toroids in equilibrium.

**Fig. 4:**
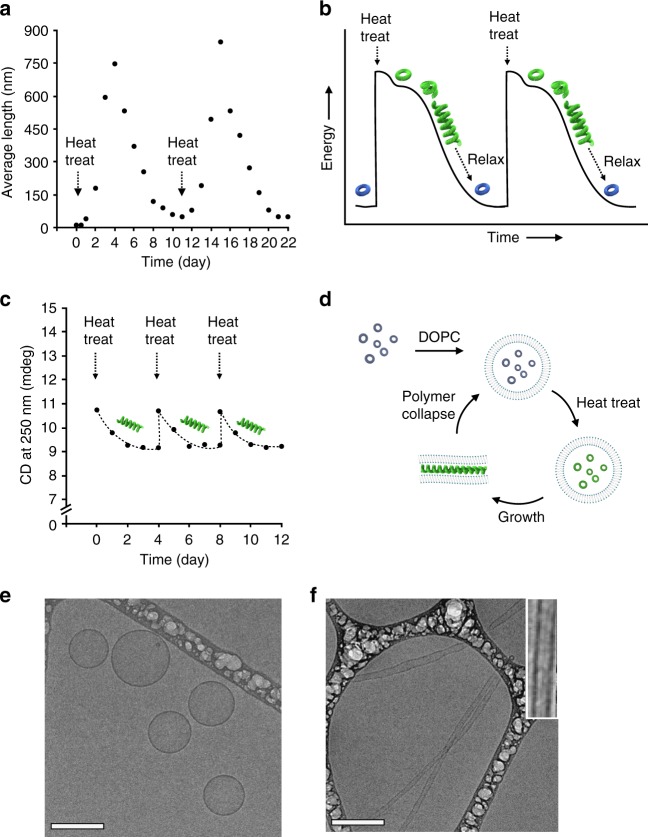
Repeated polymerization and actuation of toroid-containing vesicles. **a** Average chain lengths as a function of time, counted from cryo-TEM (*n* = 100). The subsequent heat treatments lead to identical polymerization and depolymerization cycles without sacrificing the response efficiency to energy input. **b** Energy landscape of polymerization and depolymerization cycles. **c** CD intensity (250 nm) variation of 1*S* (30 μM) in aqueous (10 vol% THF) with subsequent heat treatments at every 4 days before depolymerization undergoes. **d** Schematic representation of encapsulation of toroids inside DOPC lipid vesicles and reversible actuation of the resulting vesicles driven by polymerization-depolymerization cycle (blue, inactive toroid; green, active toroid). **e** Cryo-TEM image of vesicles including toroids after heat treatment (50 °C for 20 min) and then cooling to 25 °C. **f** Cryo-TEM image of tubular vesicles after 4 days standing (scale bar, 200 nm, inset: magnified image of representative tubular vesicles with a polymer chain inside)

Upon second heat treatment of the depolymerized toroid solution, a new cycle of polymerization undergoes to form helical chains without sacrificing the response efficiency to energy input (Fig. 4a, b, Supplementary Figures 29 and 30), demonstrating that the deactivated toroids can be repeatedly activated by subsequent heat treatments. When heat-treated every 4 days before undergoing depolymerization, the CD intensity maintains in the active state without further decay (Fig. 4c and Supplementary Figures 31-33), indicating that the helical chains exist out-of-equilibrium, which can be sustained only as far as energy is supplied.

### Reversible elongation of lipid vesicles

When the toroids are encapsulated inside lipid vesicles, the helical growth of the toroids can force vesicular walls to elongate through dimensional response, resulting in reversible shape changes of the vesicles (Fig. 4d). Encapsulation of the toroids was confirmed by tracing high-performance liquid chromatography (HPLC) after separation of the free toroids from the solution (Supplementary Figure 34). Upon heat-treated at 50 °C for 20 min and then cooled to room temperature, cryo-TEM showed spherical vesicles without structural deformation (Fig. 4e). Notably, after 4 days standing, the spherical vesicles are largely deformed into elongated tubules with a high aspect ratio (Fig. 4f), indicating that the helical growth inside the spherical vesicles forces the vesicular walls to elongate through dimensional response (Supplementary Figures 35–37), reminiscent of the cytoskeleton in cells^33^. Depolymerization is expected to drive the elongated vesicles to recover spherical shape. To shorten depolymerization time, we irradiated UV light (254 nm) to the elongated vesicles solution because UV irradiation would drive the non-planar aromatic macrocycles to undergo planarization^34^, lowering the energy barrier between slipped and eclipsed packings (Supplementary Figures 38 and 39). Indeed, when the helical chains are rapidly depolymerized by UV irradiation (over 5 min), the deformed tubular vesicles are restored immediately to its intact spherical shape (Supplementary Figures 40 and 41), indicating that the shape change of the vesicles is reversible with a polymerization–depolymerization cycle. This result demonstrates that switching into out-of-equilibrium state drives the static objects, not only to undergo autonomous polymerization/depolymerization, but also to convert external stress into mechanical work.

## Discussion

Our results show that energy supply by heat treatment can direct static objects to disclose emergent functions, capable of undergoing autonomous structural evolution, which is accompanied by performing mechanical work. This history-dependent evolution can be achieved by switching of static structures into a dynamic state using kinetic trapping of the molecular components. Such liberation from a global minimum state will pave the way towards future artificial life-like systems that have the ability to autonomously sense environmental changes and respond by performing macroscopic work.

## Methods

### General

The morphology of toroids and helical chains were characterized by TEM and AFM according to the procedures reported previously^25^. Solvent and organic reagent were purchased from commercial vendors and used without further purification unless otherwise mentioned. Analytic and preparatory HPLC were performed with Prominence LC-20AP (SHIMADZU) and YMC C8 reverse-phase columns (250 × 4.6 mm I.D. S-5 μm, 12 nm and 250 × 20.0 mm I.D. S-5 μm, 12 nm). Matrix-assisted laser desorption/ionization time of flight spectroscopy (MALDI-TOF-MS) was performed on a Bruker Microflex TOF using *trans*-2-[3-(4-*tert*-Butylphenyl)-2-methyl-2-propenylidene] malononitrile (DCTB) as a matrix. UV/Vis spectra were obtained from a Hitachi U-2900 Spectrophotometer. The fluorescence spectra were obtained from a Hitachi F-7000 fluorescence spectrophotometer. Circular dichroism (CD) spectra were obtained using Jasco J-810 spectropolarimeter. Dynamic light scattering (DLS) measurement was performed using an ALV/CGS-3.

### TEM experiments

To investigate the structures of self-assembled structures, a drop of sample solution of the dimeric macrocyle was placed on a carbon-coated copper grid (Carbon Type B (15–25 nm) on 200 mesh with Formvar; Ted Pella, Inc.) and the solution was allowed to evaporate under ambient conditions^25^. These samples were stained by depositing a drop of uranyl acetate aqueous solution (0.2–1.0 wt%) onto the surface of the sample-loaded grid. The dried specimen was observed by using a JEOL-JEM HR2100 operated at 120 kV. The cryogenic transmission electron microscopy (cryo-TEM) experiments were performed with a thin film of sample solution of dimeric macrocycle (5 µL) transferred to a lacey supported grid in room temperature (Lacey Formvar/Carbon, 200 mesh, Cu; Ted Pella, Inc.). The thin solution films were prepared under controlled temperature and humidity conditions (97–99%) within a custom-built environmental chamber to prevent evaporation of THF from sample solution. The excess liquid was blotted with filter paper for 2–3 s, and the thin solution films were rapidly vitrified by plunging them into liquid ethane (cooled by liquid nitrogen) at its freezing point. The grid was transferred, on a Gatan 626 cryoholder, using a cryo–transfer device and transferred to the JEOL-JEM HR2100 TEM. Direct imaging was carried out at a temperature of approximately −175 °C and with a 120 kV accelerating voltage, using the images acquired with a Dual vision 300 W and SC 1000 CCD camera (Gatan, Inc.; Warrendale, PA). The data were analyzed using Digital Micrograph software.

### AFM experiments

The sample films on a mica surface were prepared from evaporation of sample solutions^25^. The measurements were conducted on a MultiMode 8 AFM with NanoScope V controller, NanoScope software and NanoScope Analysis software (Bruker AXS Corporation, Santa Barbara, CA, USA) in air at ambient temperatures (ca. 25 °C) in the tapping mode. Images were acquired in PeakForce Tapping mode.

### NMR experiments

The peak assignment in folded conformation was confirmed by 2D ROE experiments. 1*S* (30 μM) in D_2_O/THF-*d*_8_ (1/1, v/v) was sonicated for 30 min. The sample solution was kept for 4 h at room temperature for stabilization, before 2D ROE spectra were measured on a 600 MHz FT NMR spectrometer. To understand the relationship between CD increment and packing change of the dimeric macrocycle, we performed NOE experiments with addition of KF salt into the toroid solution because the oligoether chains undergo salting-out effect with KF salt, leading to hydrophobic collapse, similar to LCST effect. Different from the heat-treated solution, the KF solution provides highly-resolved NOE-NMR data to obtain the information on packing mode of the dimeric macrocycles. The twist stacking of dimeric macrocycles was recorded using a solution of 1*S* (300 μM) in D_2_O/THF-*d*_8_ (1/1, v/v) with different concentration of KF with sonication for 30 min and stabilized for 4 h. One-dimensional (1D) nuclear overhauser effect (NOE) measurements were performed to gain more insight into the aromatic interactions. A ^1^H resonance is selectively irradiated until saturation is achieved. During the irradiation period, NOE buildup occurred at other ^1^H nuclei close in space and were detected. The experiments were repeated using different irradiation frequencies. The integrations of correlation peaks were normalized through irradiation peaks.

### Molecular dynamics simulations

Molecular structures of eclipsed and slipped packing modes were discovered by all-atom (AA) molecular dynamics (MD) simulations. The AA MD simulations employed AMBER force field with the partial charges of atoms determined by R.E.D. (RESP ESP charge Derive) after the structure optimization using M062x with 6–31 g (d,p) basis set implanted in Gaussin09. The water molecule is modeled by TIP3P. The AA (MD) simulations were performed by NAMD package with isothermal-isobaric (NPT) ensemble under one bar pressure. The energy diagram of toroids before and after heat treatment and the equilibrium constant of opening toroids were derived from large-scale coarse-grained (CG) MD simulations in which a group of atoms in similar size are taken as a CG particle. To be specific, the phenyl group and the oligoether chain segment with five heavy atoms including carbon and oxygen are represented by CG particles. All CG simulations were carried out by GALAMOST package. The aromatic structural changes under UV irradiation was identified by quantum chemistry calculation using M06l with 6–31 g (d,p) basis set implanted in Gaussin09. The polymerization mechanism was verified by kinetic Monte Carlo simulations.

### Vesicle preparation

Lipid films were prepared by rotary evaporation of DOPC (10 mg) in methanol. All subsequent steps were performed at room temperature. The lipid film was rehydrated with 2 ml of aqueous (10 vol% THF) solution for 1 h. A 2 ml toroids aqueous (10 vol% THF) solution of 1*S* (300 μM) was added to lipid solution. The resulting vesicle suspension was extruded 15 times through a polycarbonate Track-Etch membrane (pore size 0.5 μm) using an Avanti Mini Extruder (Avanti Polar Lipids, Inc). After extrusion, the vesicle was purified by sephacryl S-500 HR column (eluent: H_2_O with 10 vol% THF, flow rate: 0.2 ml/min) to remove free toroid. The collected fraction was heated into 50 °C for 20 min and stored at room temperature. Control experiments were performed by mixing the toroids solution and DOPC vesicle solution without encapsulation of toroids. The mixture solution was heated into 50 °C for 20 min without sephacryl purification and stored at room temperature.

### UV irradiation experiments

Polymer solution of 1*S* in aqueous (10 vol% THF) was transferred to an ultraviolet quartz cuvette, then sealed with a screw cap^35^. Depolymerization was conducted under 254 nm ultraviolet light exposure, the solution was monitored by TEM and CD spectropolarimeter after 5 min.

## Supplementary information

Supplementary Information

Description of Additional Supplementary Files

Supplementary Movie 1

Supplementary Movie 2

## Data Availability

The data that support the findings of this study are available from the corresponding author upon reasonable request.
