# Supplementary material for: Autonomous helical propagation of active toroids with mechanical action
Source: Nat Commun. 2019 Mar 6;10:1080. doi: 10.1038/s41467-019-09099-9 (PMC6403424; doi:10.1038/s41467-019-09099-9)
Supplement: Supplementary file 1 — Supplementary Information [file 41467_2019_9099_MOESM1_ESM.pdf]

Supplementary Information for

Autonomous Helical Propagation of Active Toroids with Mechanical Action

Bowen Shen *et. al.*

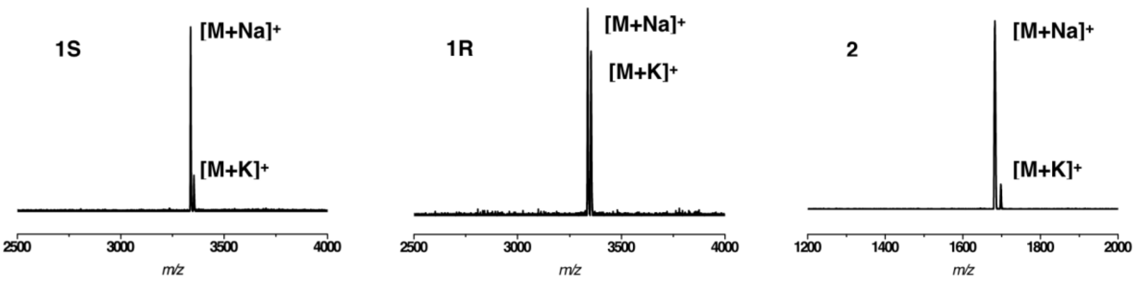

Supplementary Figure 1. MALDI-TOF mass spectra of molecule **1*S***, **1*R*** and **2**.

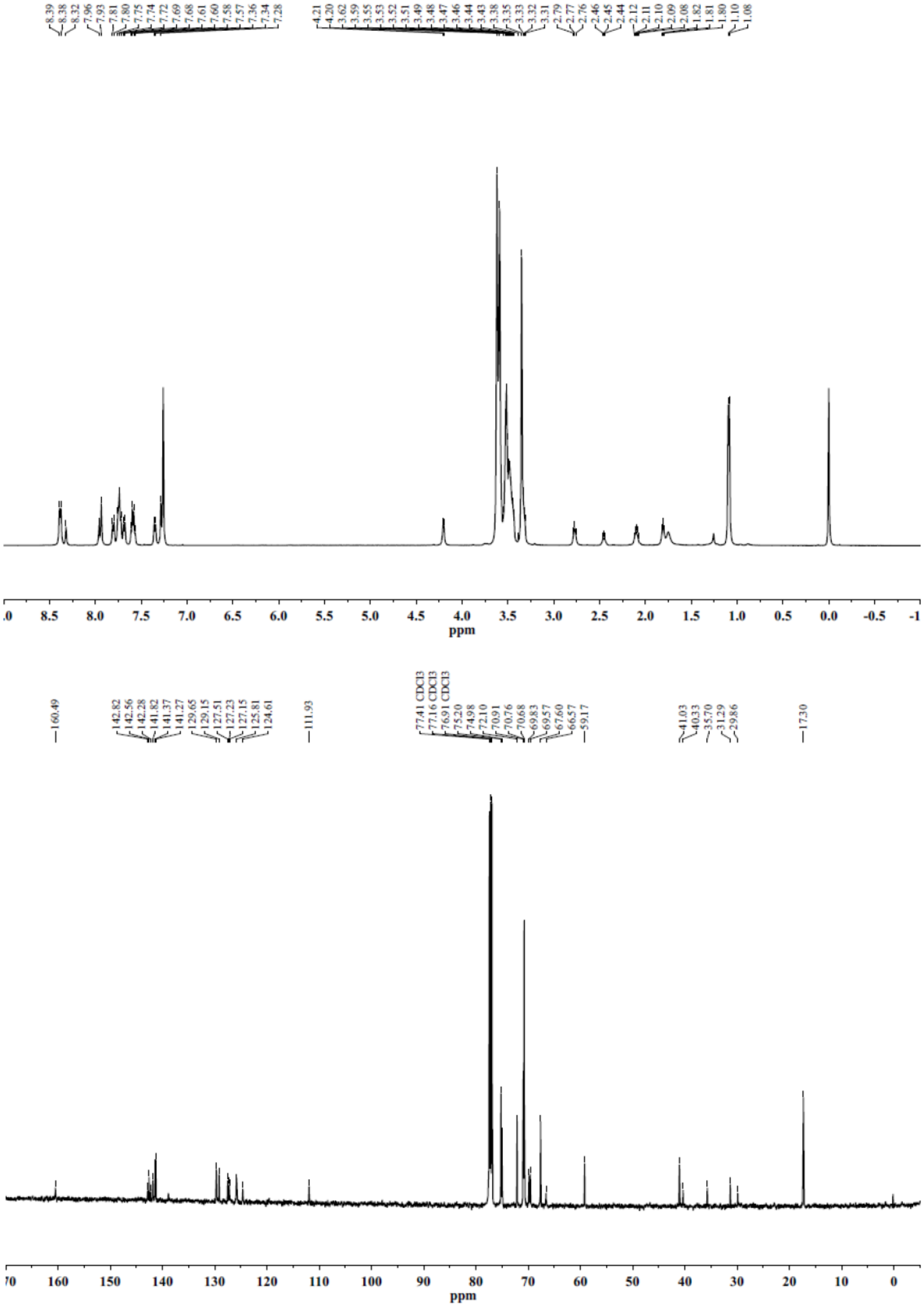

Supplementary Figure 2. $^{1}H/^{13}C$ NMR spectra of **1** in $CDCl_3$

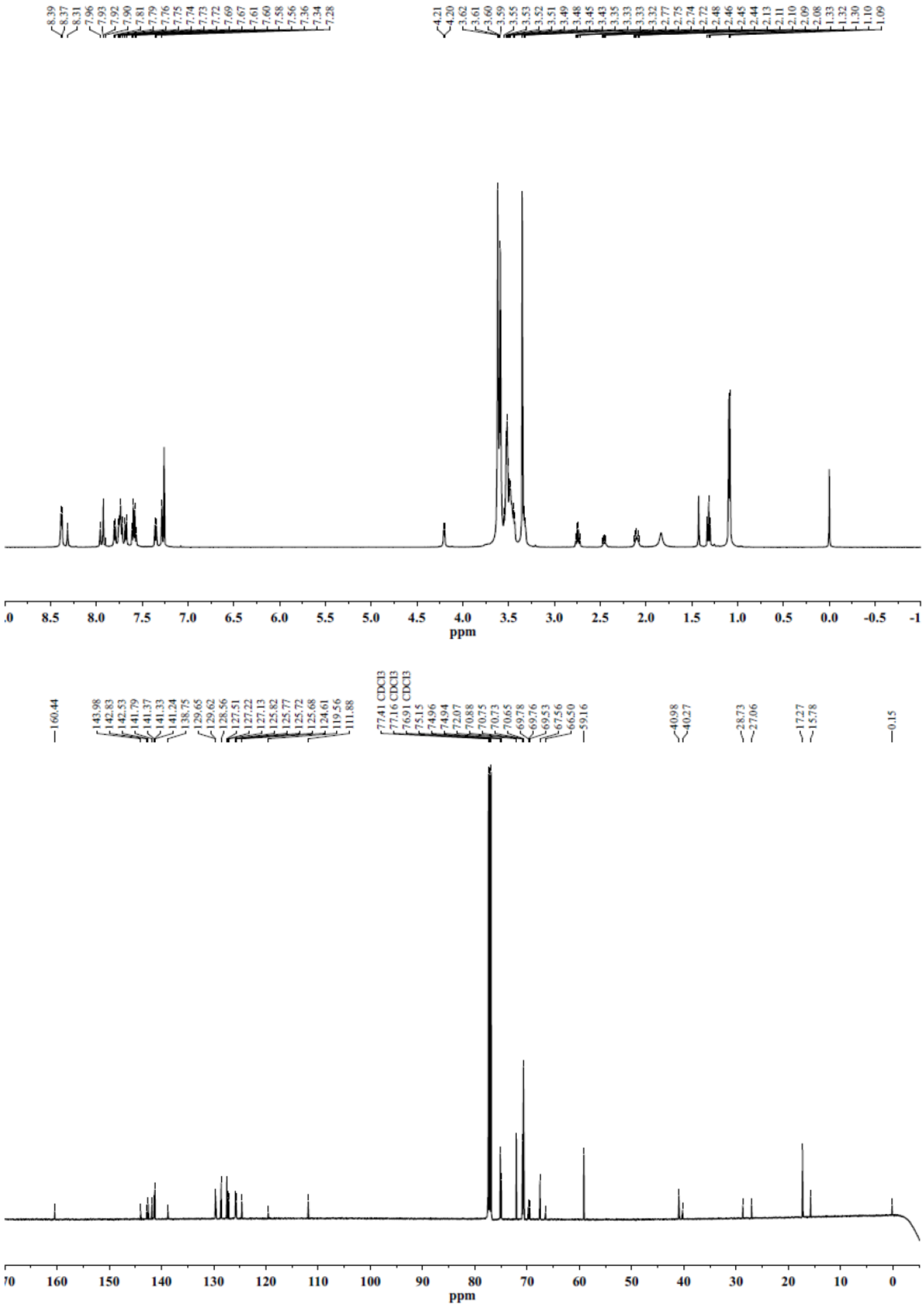

Supplementary Figure 3. $^{1}H/^{13}C$ NMR spectra of **2** in $CDCl_3$

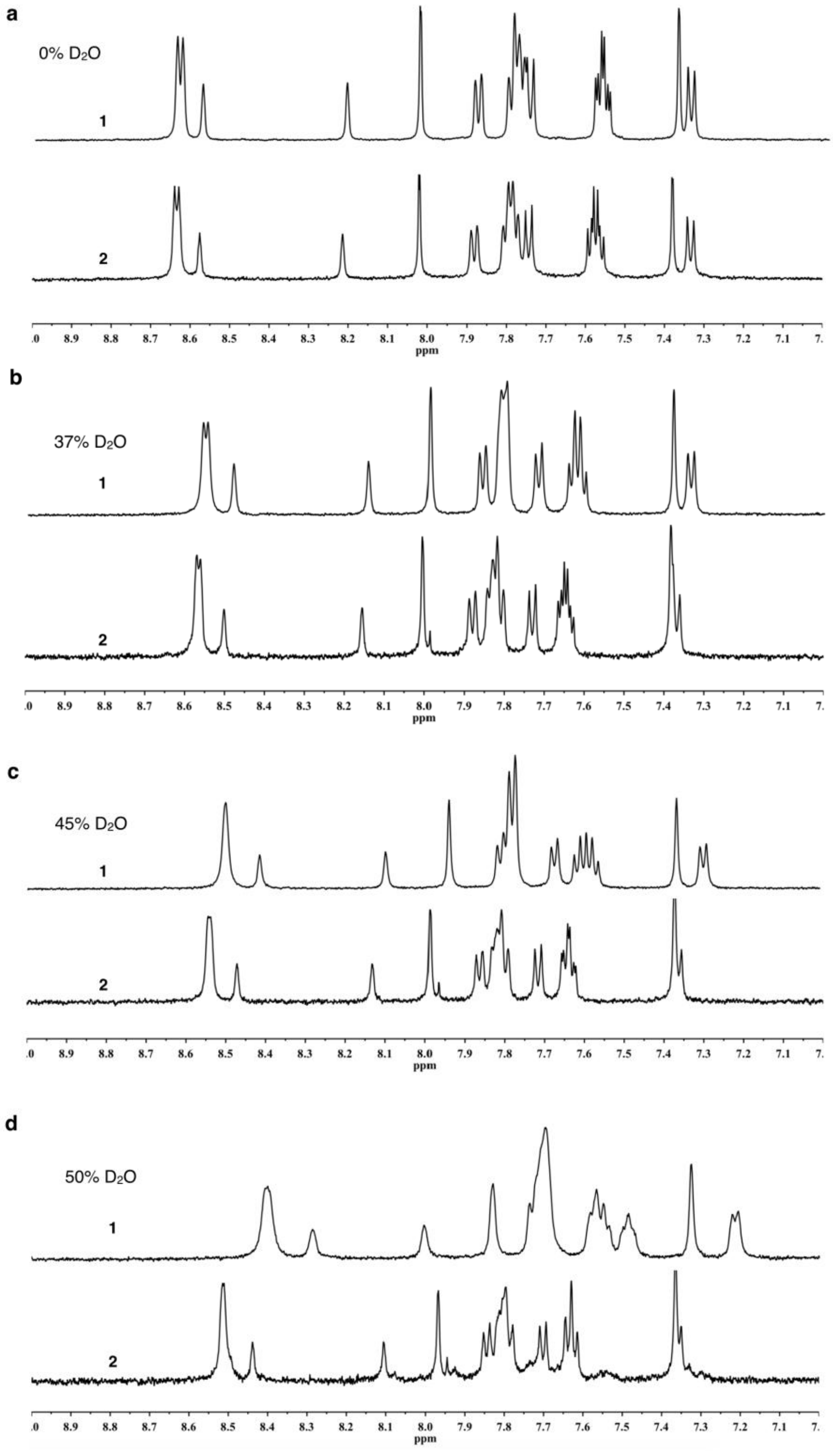

Supplementary Figure 4. ^{1}H-NMR spectra of molecule **1** and **2** (30 μM) in THF-d_8 with increasing the D_2O content, (a) 0% D_2O, (b) 37% D_2O, (c) 45% D_2O and (d) 50% D_2O. The up field shift of **1** compared with the monomeric model molecule **2**, indicating the folded conformation of the aromatic segment of **1** in D_2O/THF-d_8 (1/1, v/v) solvent condition.

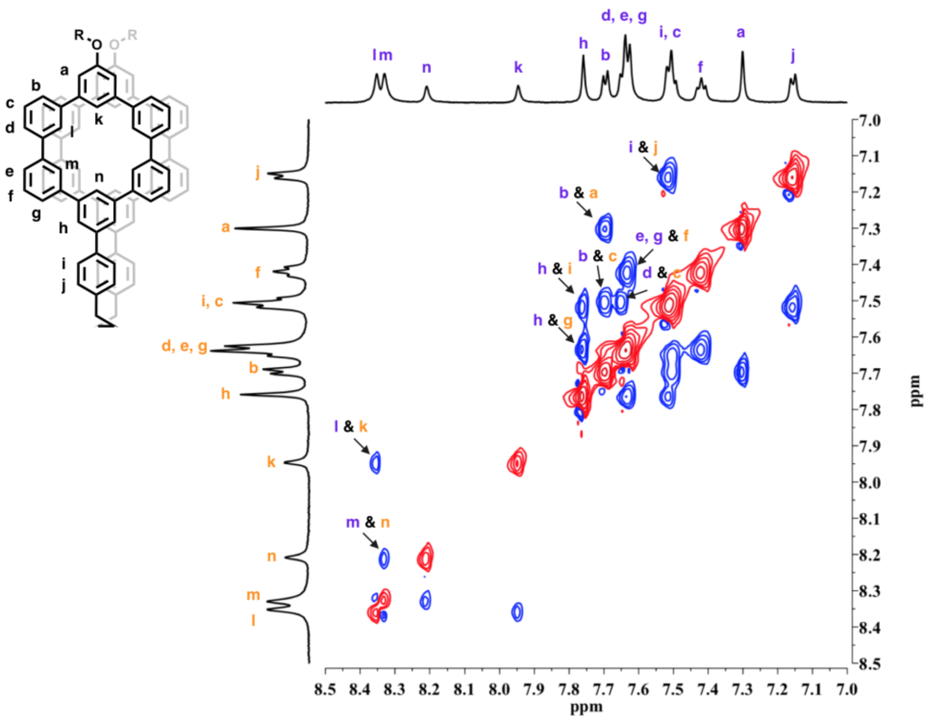

Supplementary Figure 5. Aromatic segment peak assignment from 2-dimensional rotating-frame overhauser effect (ROE) NMR spectrum of **1*S*** (300 μM) in D_2O/THF-d_8 (1/1, v/v) in which molecule **1*S*** was believed to be folded conformation.

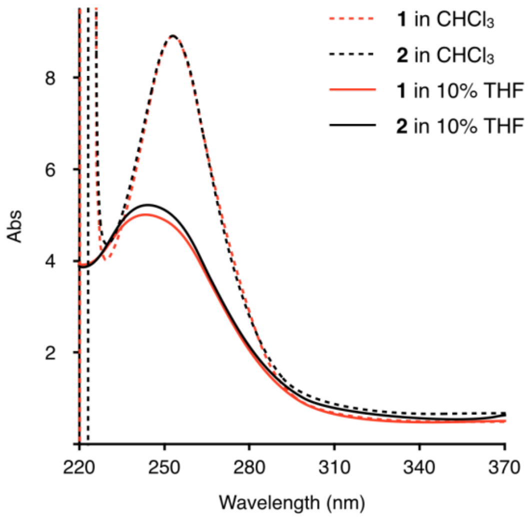

Supplementary Figure 6. Absorption spectra of molecule **1** (30 μM) and **2** (60 μM) in chloroform (**1**: red and dashed line, **2**: black and dashed line) and in aqueous (10 vol% THF) solution (**1**: red and solid line, **2**: black and solid line). Compared with **2**, molecule **1** shows an absorption decrease in aqueous (10 vol% THF) solution, indicating **1** adopts folded conformation.

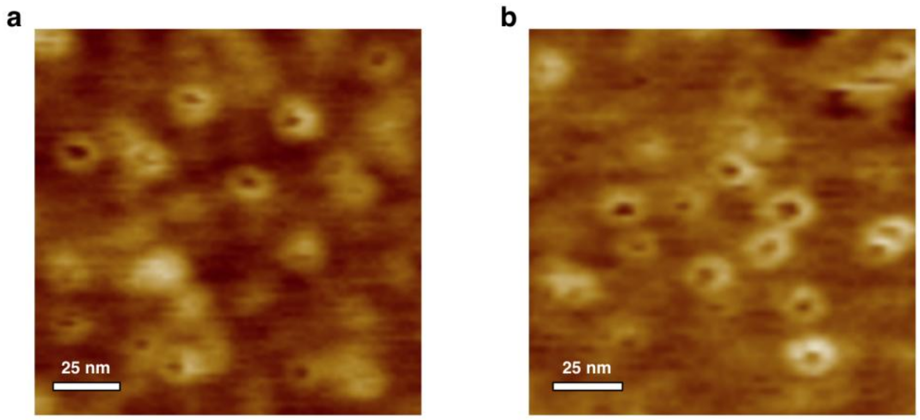

Supplementary Figure 7. AFM phase image of the film on a mica surface from evaporation of **1*S*** (30 μM) in aqueous (10 vol% THF) solution before (a) and after heat treatment (b), toroidal structures remained unchanged after heat treatment.

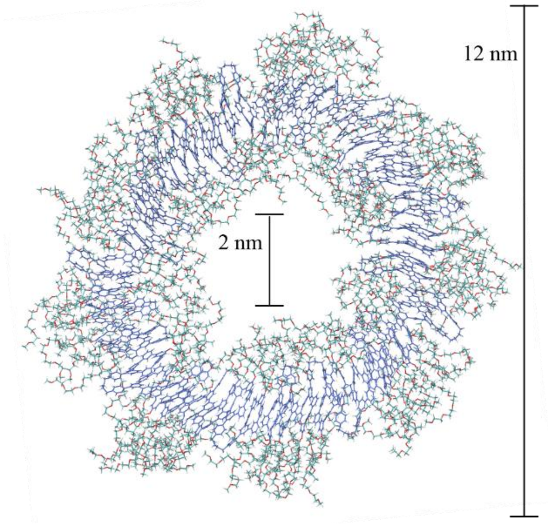

Supplementary Figure 8. The snapshot of inactive toroid with molecules in eclipsed conformation obtained in AAMD simulations at 100 ns. The toroid with a number of 27 constituent molecules has a comparable size of 12 nm to the experimental value. Most importantly, the packed structure could be stably kept in long-time AAMD simulation (simulation time: 100 ns, Supplementary Movie 1). After a relaxation period of AAMD simulations, the wedge-shaped molecules with dimeric aromatic core in eclipsed state are aligned roughly perpendicular to the plane of the toroid and the hydrophobic aromatic cores of toroid are surrounded by oligoether chains.

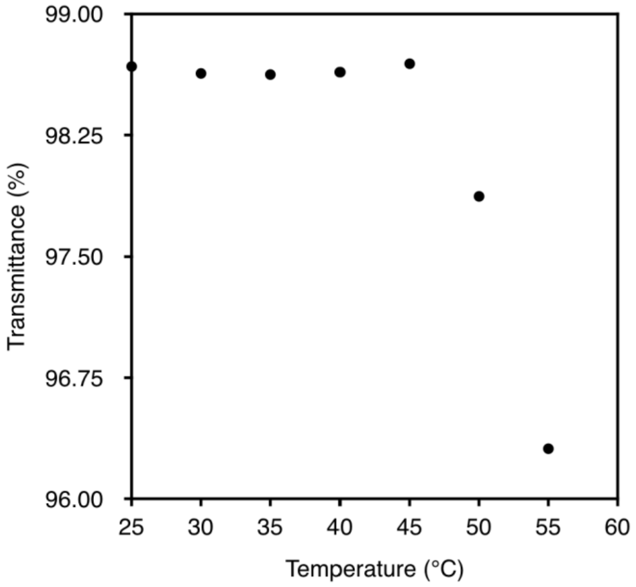

Supplementary Figure 9. Temperature-dependent transmittance of **1*S*** (30 μM) in aqueous (10 vol% THF) solution, indicating that the oligoether dendrons are dehydrated into globular conformation upon heating.

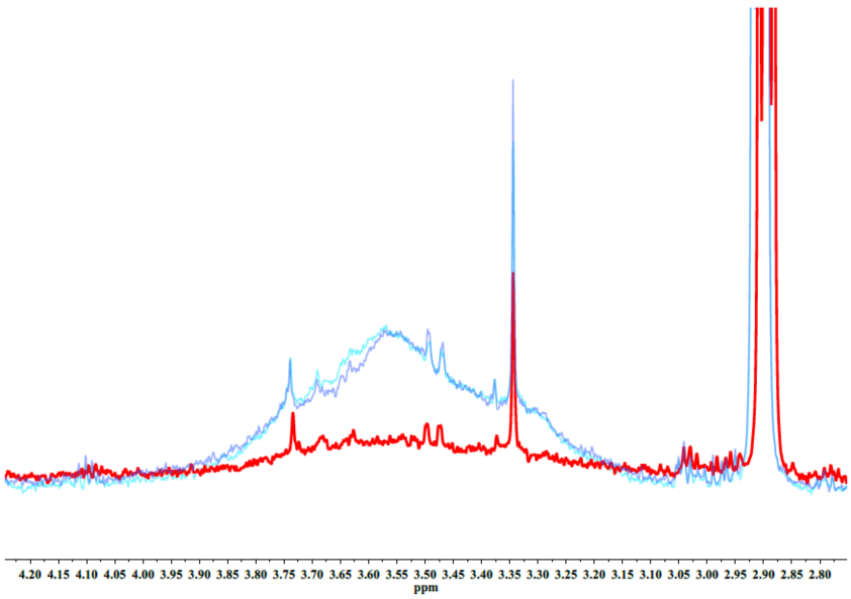

Supplementary Figure 10. Temperature-dependent ^{1}H-NMR spectra of **1*S*** (30 μM) in D_2O, at 25 ℃ (light blue), 50 ℃ (red) and cooling to 25 ℃ (blue). The figure shows that the reversible peak broadening of resonances (3.1 ppm - 4.0 ppm) associated with oligoether dendrons, indicating the oligoether dendrons switch to globular conformation by thermal dehydration, and completely rehydrate after cooling down.

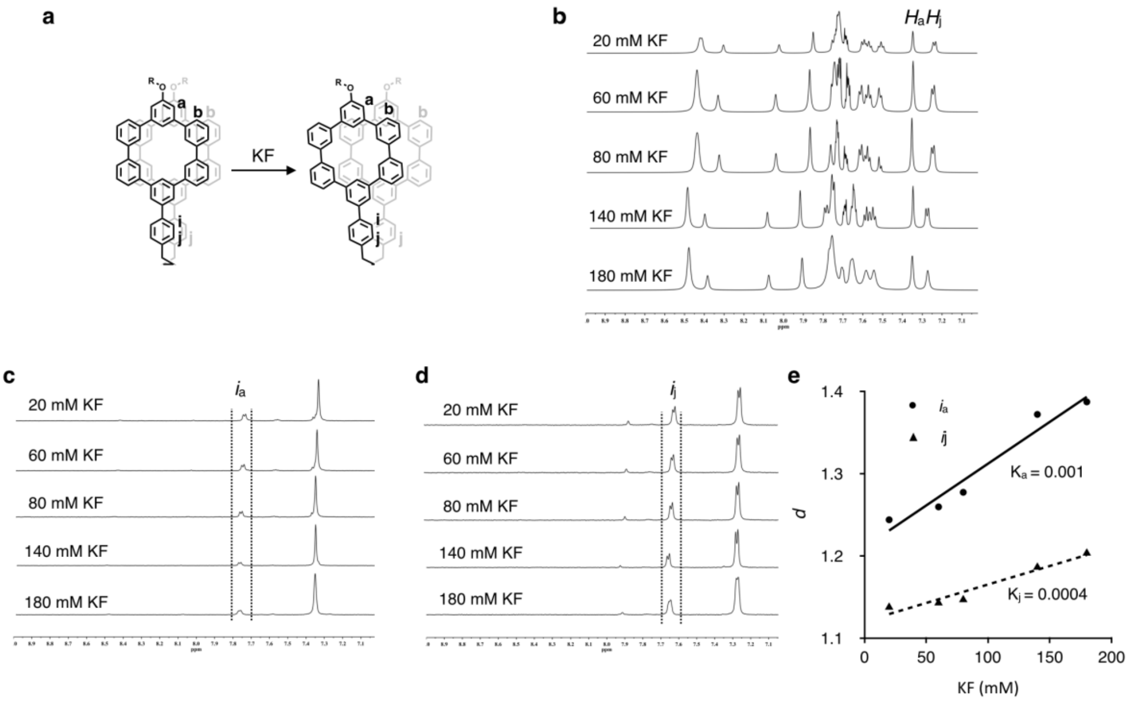

Supplementary Figure 11. 1D NOE spectra of **1*S*** (300 μM) in D_2O/THF-*d8* (1/1, v/v) with a series of KF concentrations. (a) Schematic representation of salt effect induces slipped conformation of aromatic macrocycle **1**. (b) ^{1}H-NMR spectra of aromatic segment of **1*S*** (300 μM) in D_2O/THF-*d8* (1/1, v/v) with different concentrations of KF. (c) 1D NOE spectra of irradiation at 7.38-7.31 ppm (corresponding to H_a) with KF induced NOE correlations with H_b at 7.78-7.72 ppm. (d) 1D NOE spectra of irradiation at 7.31-7.19 ppm (corresponding to H_j) with KF induced NOE correlations with H_i at 7.68-7.56 ppm. (e) NOE correlations peak integrations (*i*) as a function of KF concentration, *d* (*d*=1/ [(*i*) ^1/6]) representing the distance between H_a & H_b or H_j & H_i. By linear fitting, the slop of H_a & H_b (K_a, solid line) shows sharper than H_j & H_i (K_j, dash line), the results indicate that H_a & H_b shows greater displacement, revealing the slipping conformation of dimeric macrocycle **1**. The NOE titrations showed that the addition of KF induces a slipped packing arrangement due to the hydrophobic collapse of the dendritic chains of **1*S*,** consistent with the CD increment when add KF (Supplementary Fig. 12). Considering the hydrophobic collapse of the dendritic chains is also followed by heat-treatment, this result supports that the CD increment followed by LCST arises from the same slipped packing of the dimeric macrocycle.

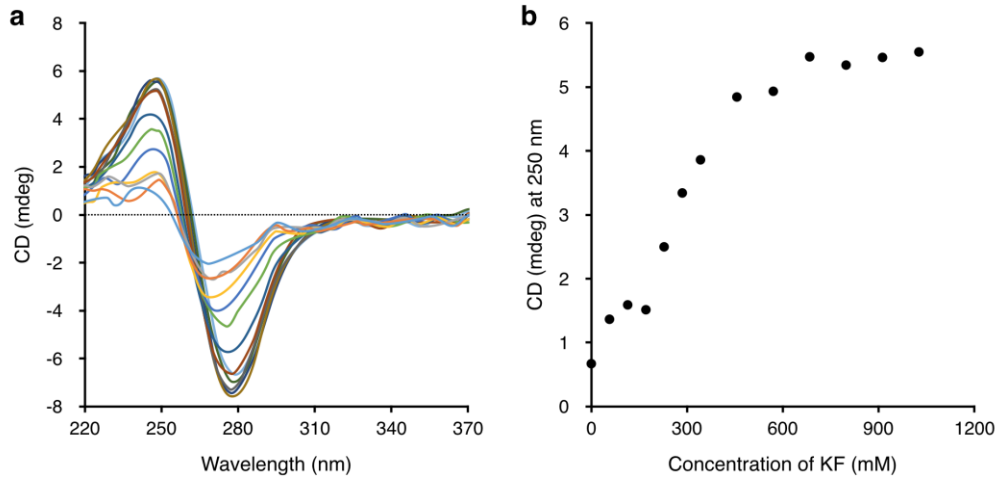

Supplementary Figure 12. CD spectra (a) and intensities at 250 nm (b) of **1*S*** (30 μM) in aqueous (20 vol% THF) solution with addition of KF. Addition KF into the **1*S*** solution also induces the same CD increment as heat treatment, indicating that KF plays an identical role in CD increment to LCST.

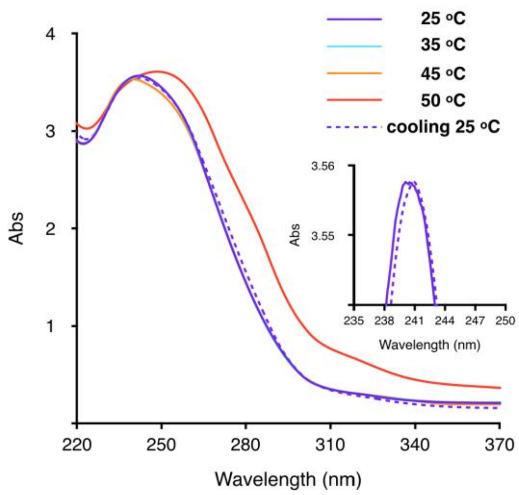

Supplementary Figure 13. Temperature-dependent absorption spectra of molecule **1*S*** (30 μM) in aqueous (10 vol% THF) solution. The red shift of absorption at 50 ℃ suggests the slipped packing of aromatic segment with thermal dehydration of oligoether dendrons. After heat treatment, the absoption showed 1 nm red shift, indicating that the macrocycles were trapped in slipped conformation.

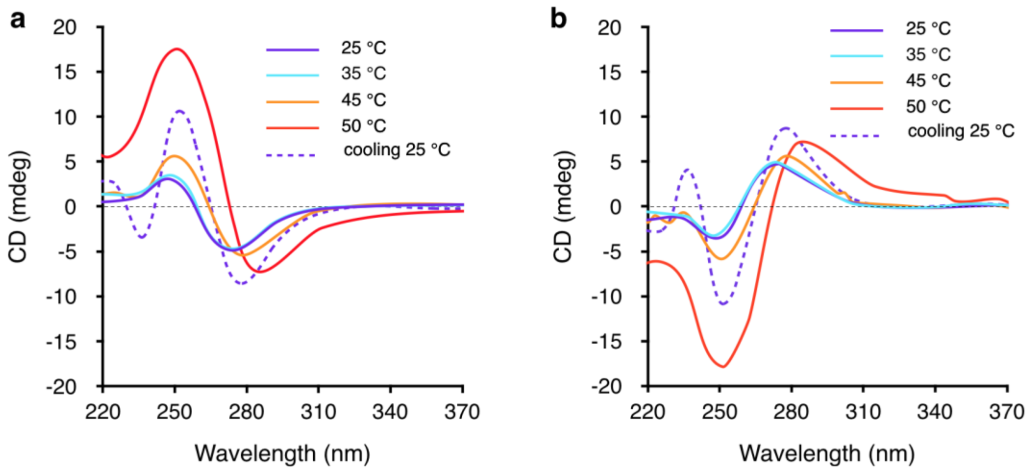

Supplementary Figure 14. Temperature-dependent CD spectra of molecule (a) **1*S*** and (b) **1*R*** from 30 μM in aqueous (10 vol% THF) solution. **1*R*** shows a mirror image relationship with **1*S***, indicating the chiral transfer from the asymmetric centers in the dendritic chains.

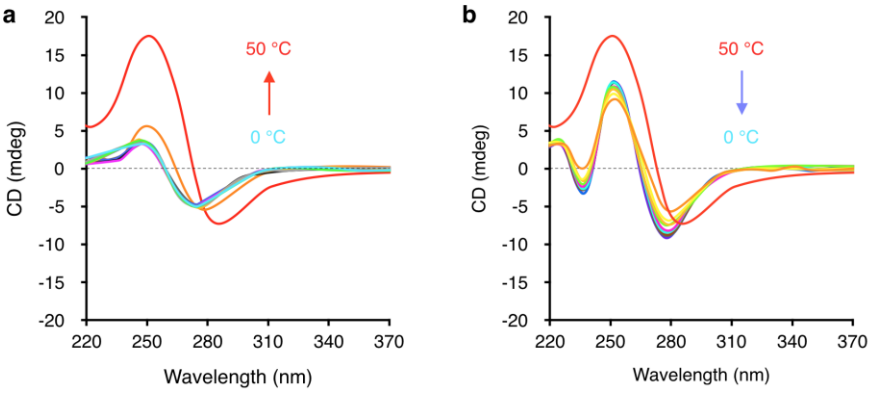

Supplementary Figure 15. (a) Temperature-dependent CD spectra of molecule **1*S*** (30 μM) in aqueous (10 vol% THF) solution heated from 0 ℃ to 50 ℃, and (b) cooled from 50 ℃ to 0 ℃.

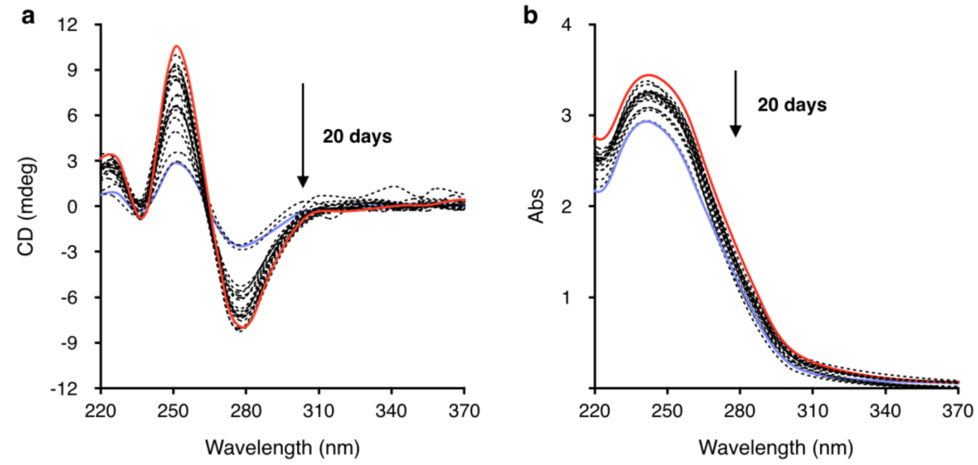

Supplementary Figure 16. (a) CD spectra decayed and absorption spectra (b) at room temperature of **1*S*** (30 μM) in aqueous (10 vol% THF) solution after heat treatment (50 °C for 20 min).

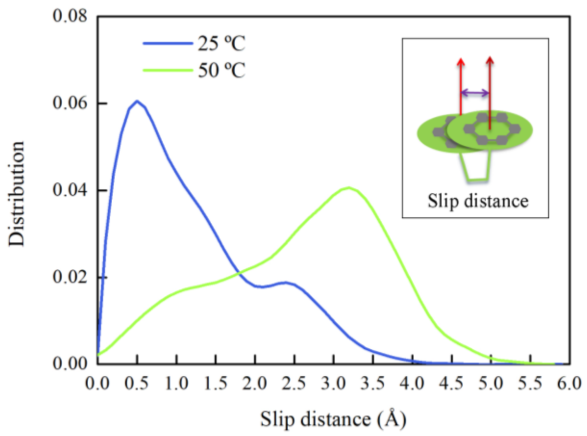

Supplementary Figure 17. Slip distance distribution of dimeric aromatic macrocycle at 25 °C (blue) and 50 °C (green) by AAMD simulation (simulation time: 150 ns). At 25 °C, the molecule has slipped (the slip distance is 3.5 Å) and eclipsed (0.5 Å) packing states. The molecular configurations can change between these two packing states with time. From simulation trajectory, we could identify that the aromatic segment resides in eclipsed conformation for about 80% of whole time. The free energy of slipped conformation is higher than eclipsed one with about 3.5 kJ/mol, which is calculated by $\Delta G = -RT\ln(K)$ with $K = 1/4$ and $T = 298$ K. As the temperature increases to 50 °C, the free energy of eclipsed conformation is higher than slipped one with about 4.2 kJ/mol. The time for aromatic segment residing in slipped conformation becomes larger, indicating dimeric aromatic macrocycle prefers switching into slipped conformation at 50 °C.

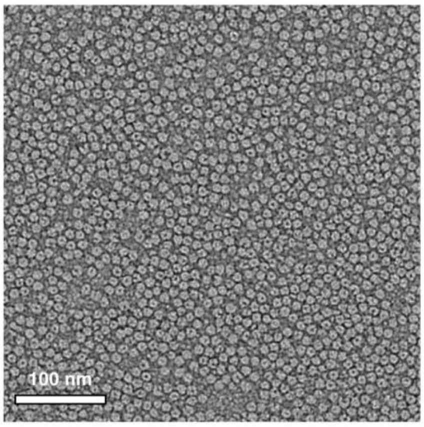

Supplementary Figure 18. Negatively-stained TEM image of **1*S*** (30 μM) in aqueous (10 vol% THF) solution after heat treatment (50 ℃ for 20 min), and then cooled to room temperature to stand for 10 min, no helical chains were observed.

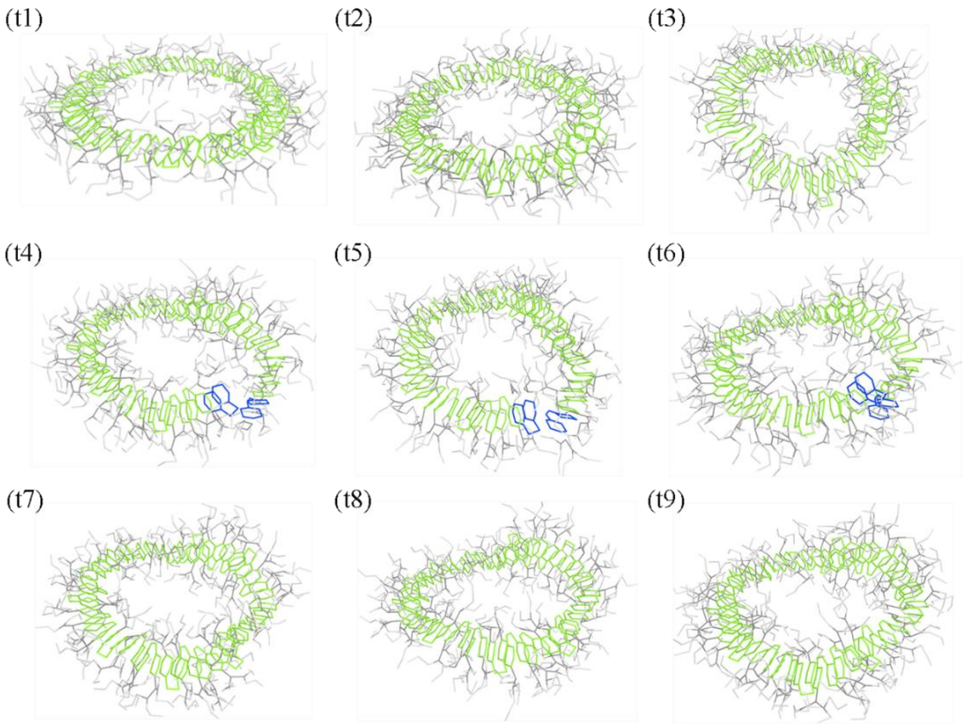

Supplementary Figure 19. The spirally opening of active toroid by CGMD simulation. The molecules at open ends of the toroid are colored blue. The active toroids exhibit dynamic equilibrium between closed form and spirally open form, which can be observed in CGMD simulations (simulation time: 16 μs, Supplementary Movie 2 showed 100 ns). In the beginning of simulations, molecular aromatic discs in toroid were tilted with respect to the plane normal to keep the planar and circular shape of the toroid. As time going, the exposure of aromatic discs to water environment which imposes a strain on the contour of toroid drives it to be distorted and unstable. From time to time, the toroid will be spirally open. However, the high energy of open toroid with two ends will help the toroid transform back to closed structure. The dynamic toroid resides in closed state in most of the time, and the residing time ratio 5.5 x 10^{-3} between open state and closed state can be estimated by the equilibrium constant calculated from the energy difference as introduced in Supplementary Figure 28.

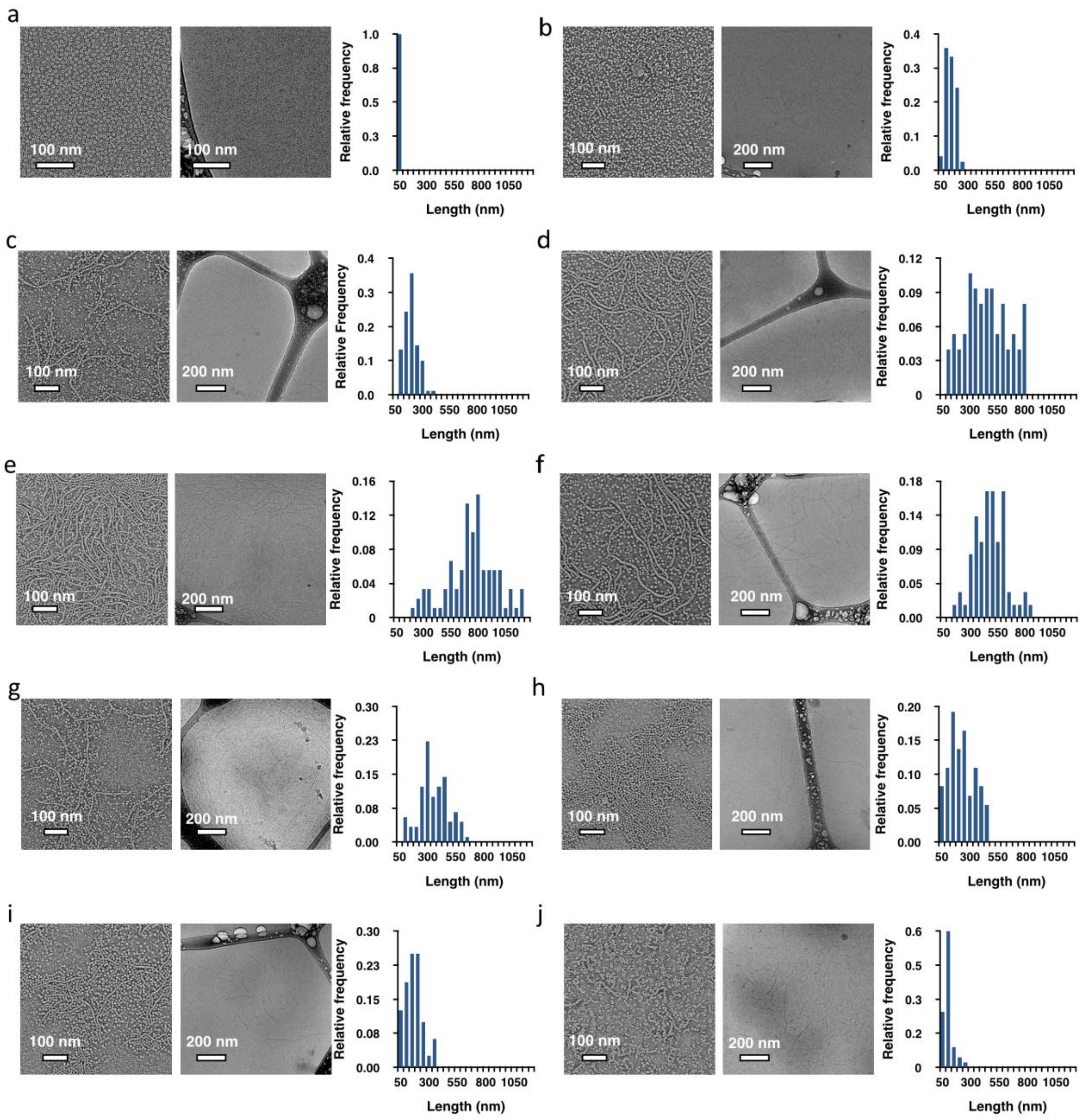

Supplementary Figure 20. Time-dependent negatively stained TEM (left), cryo-TEM images (middle) and histograms of the length distribution (right), counted from the cryo-TEM images (n=100), of **1*S*** (30 μM) in aqueous (10 vol% THF) solution after heat treatment (50 ℃ for 20 min) and then standing at room temperature. (a) 12 h, (b) 1 day, (c) 2 days, (d) 3 days, (e) 4 days , (f) 5 days, (g) 6 days, (h) 7 days, (i) 8 days and (j) 9 days.

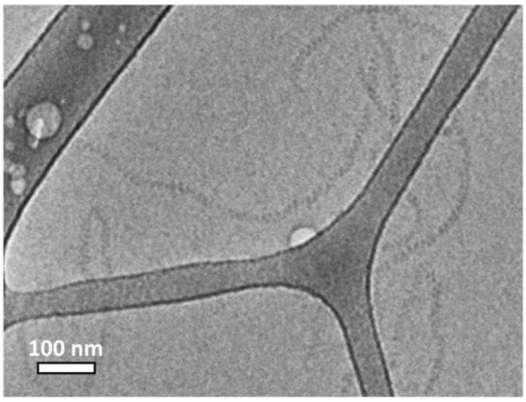

Supplementary Figure 21. Magnified cryo-TEM image of helical chains from **1*S*** (30 μM) in aqueous (10 vol% THF) solution after heat treatment (50 ℃ for 20 min), and then cooled to room temperature to stand for 4 days. The helical array of aromatic core was observed, which excludes toroidal stacking.

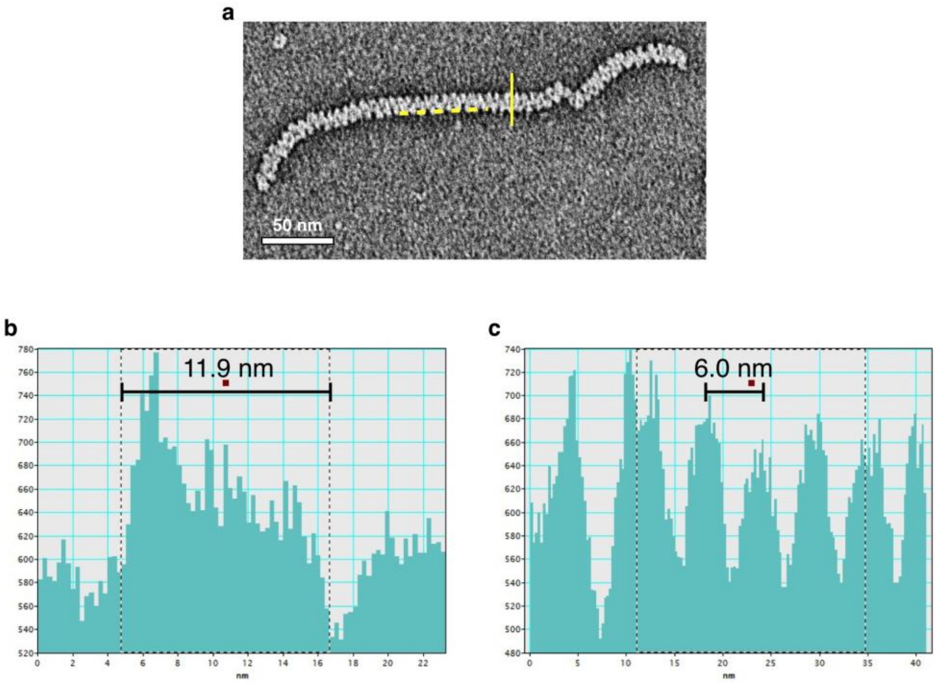

Supplementary Figure 22. (a) Magnified negatively-stained TEM image of a helical chain from **1*S*** (30 μM) in aqueous (10 vol% THF) solution after heat treatment (50 ℃ for 20 min), and then cooled to room temperature to stand for 4 days. Electron density profiles denoted by the (b) solid line and (c) dash line, suggesting the detailed diameter (12 nm) and pitch length (6 nm) of the helical chain.

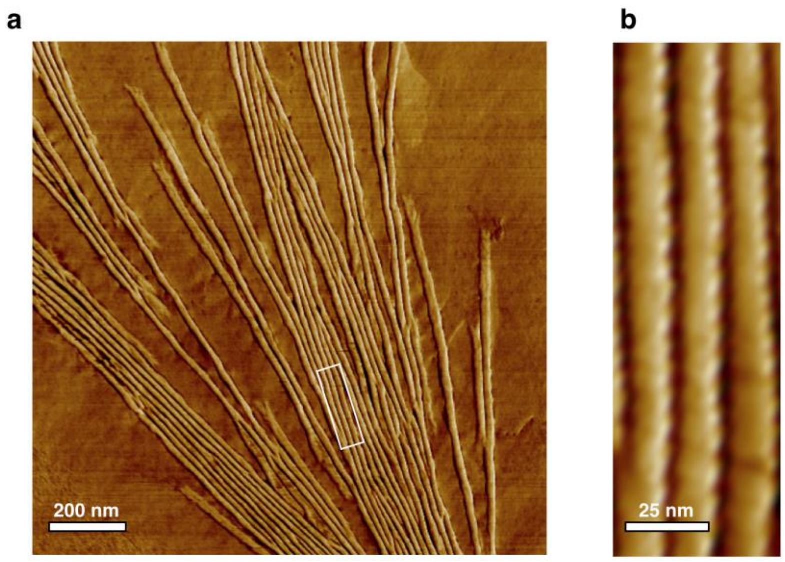

Supplementary Figure 23. (a) AFM phase image of the film on a mica surface from evaporation of **1*S*** (30 μM) aqueous (10 vol% THF) solution after heat treatment (50 ℃ for 20 min) and then standing 4 days at room temperature, (b) the magnified image corresponding to the area indicated by the white square.

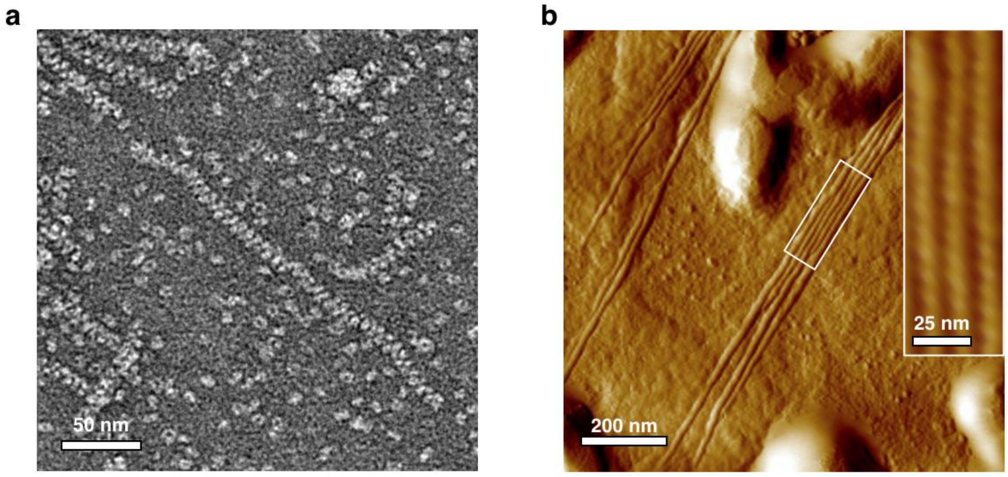

Supplementary Figure 24. (a) Negatively-stained TEM and (b) AFM phase images of **1*R*** (30 μM) aqueous (10 vol% THF) solution after heat treatment (50 ℃ for 20 min) and then standing 4 days at room temperature, inset was magnified image corresponding to the area indicated by the white square. Left-handed helical chains were observed.

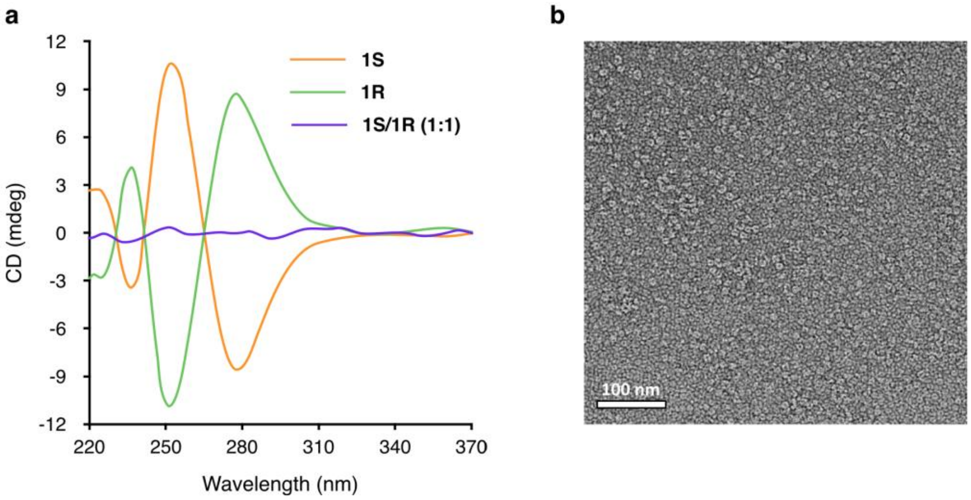

Supplementary Figure 25. (a) CD spectra of **1*S*** (orange), **1*R*** (green) and **1*S*/1*R*** co-assembly (purple) (30 μM) in aqueous (10 vol% THF) solution after heat treatment (50 ℃ for 20 min). (b) Negatively-stained TEM image of **1*S*/1*R*** co-assembly (30 μM) in 10% THF (v/v) aqueous solution after heat treatment (50 ℃ for 20 min) and then standing 4 days at room temperature, no helical chains were observed. The co-assembly solution was prepared by evaporation of a $CHCl_3$ mixture of **1*S*** and **1*R*** (mol/mol, 1/1), then 10% THF (v/v) aqueous solution was added to the dry film and the solution was sonicated for 30 min in ice bath and standing at least 4 hours at room temperature.

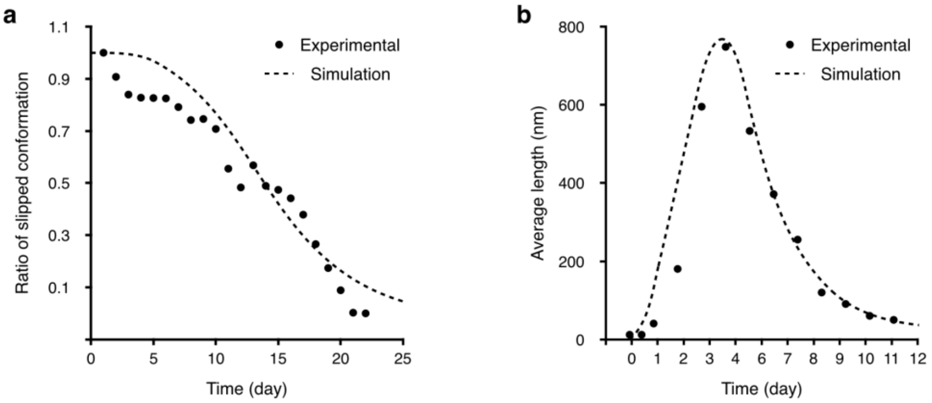

Supplementary Figure 26. Kinetic Monte Carlo simulation fitting curves. (a) The decaying rate of slipped molecules was estimated from CD spectra (cf. Figure 3e in main text). (b) The fitting curve of polymerization/depolymerization of active toroids.

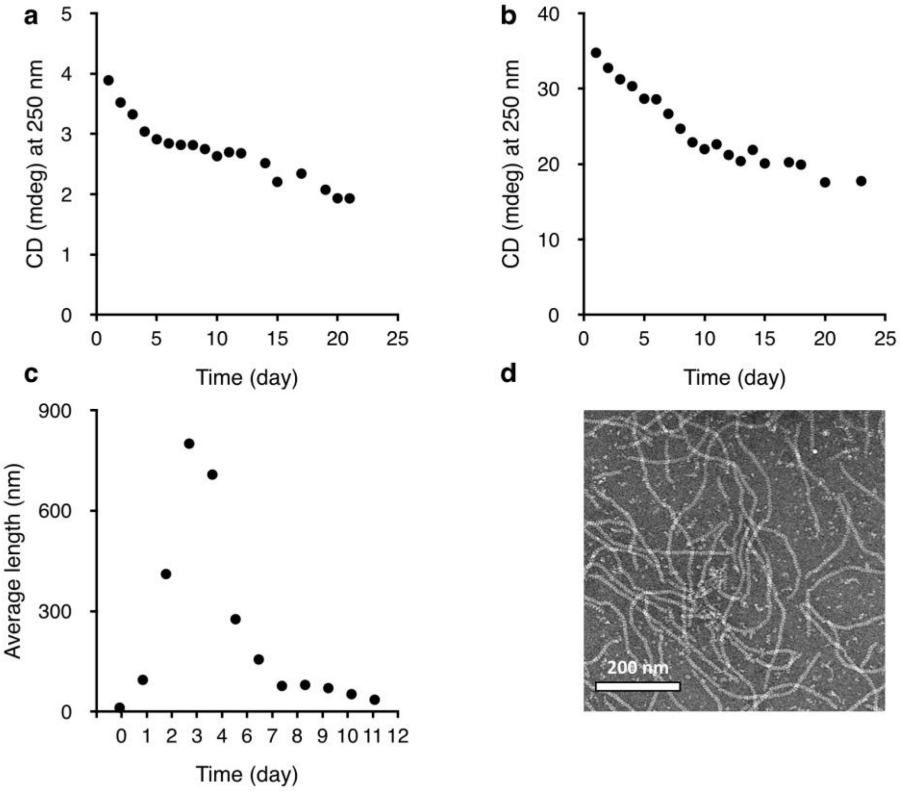

Supplementary Figure 27. Polymerization/depolymerization kinetics for different concentration of **1*S***. Time-dependent CD intensities at 250 nm from 10 μM (a) and 90 μM (b) of **1*S*** solution. (c) Time-dependent average chain lengths from 90 μM solution, counted from negatively-stained TEM images (n=100). (d) Negatively-stained TEM of polymers from 90 μM solution at 3 days.

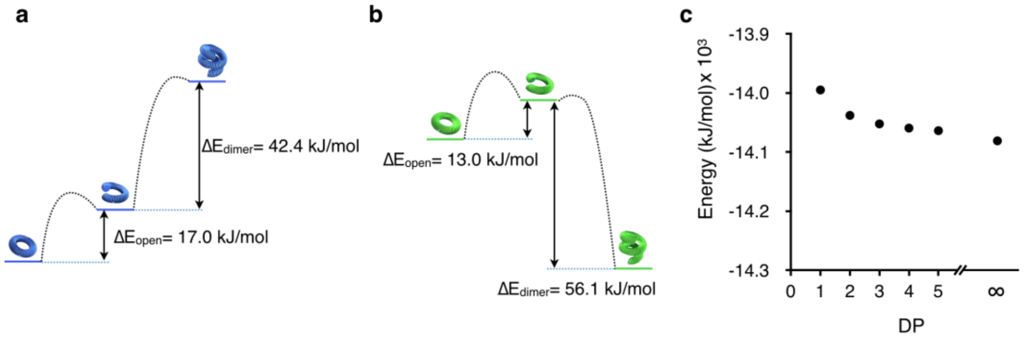

Supplementary Figure 28. Energy diagrams for the spirally opening and dimerization of toroids based on (a) eclipsed and (b) slipped conformation. (c) Energy calculation of polymerization by CGMD simulation. The energy of polymerized helical structure could also be predicted by $E_N = E_0 - 2\Delta E(N\text{-}1)/N$, where N is the toroid number in a polymerized helical structure, E_0 is the energy of closed toroid, and ΔE is the energy difference of two closed toroids to a helical toroid dimer. The calculation results of oligomers from simulations are consistent with the ones predicted by the theoretical formula. The energy of infinitely long polymer thereby is $E_{\text{infinite long}} \approx E_0 - 2\Delta E$. The energy of open toroid is higher than that of closed toroid in molecular slipped packing mode. However, the energy difference is so small and could be overcome by thermal fluctuation accompanied by the transformation from closed toroid to open toroid. The equilibrium constant of open toroid to closed toroid could be estimated by $K = \exp(-\Delta G/RT) \approx \exp(-\Delta H/RT) = 0.0055$ with $\Delta H = 13.04$ kJ/mol. The dynamic transformation between closed toroid and open toroid could also be directly observed in CGMD simulations. The proportion of residing time between open toroid and closed toroid from CGMD simulations is closed to the value calculated from energy.

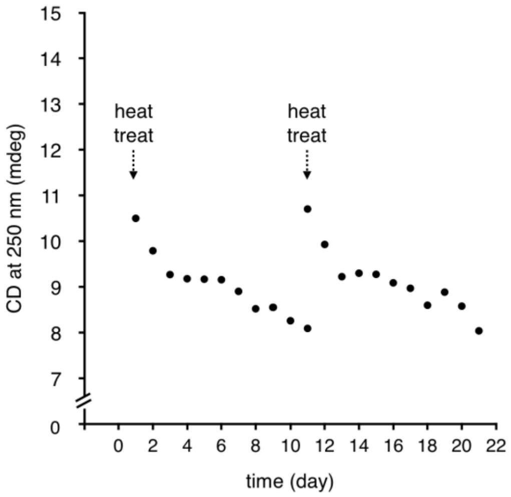

Supplementary Figure 29. CD intensities (250 nm) as a function of time of **1*S*** (30μM) at aqueous (10 vol% THF) solution. The subsequent heat treatments lead to identical cycles, indicating the repeatable polymerization and depolymerization.

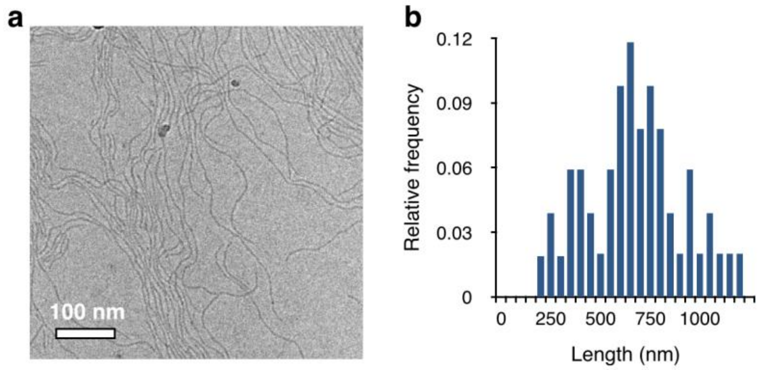

Supplementary Figure 30. (a) Cryo-TEM image and (b) histogram of the length distribution, counted from cryo-TEM images (n=100), of **1*S*** (30μM) at aqueous (10 vol% THF) solution after 2nd heat treatment and then standing 4 days at room temperature.

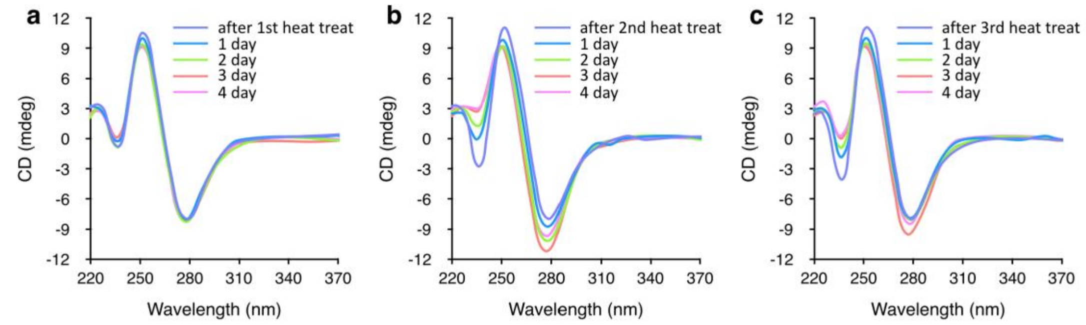

Supplementary Figure 31. Time-dependent CD spectra of **1*S*** (30 μM) in aqueous (10 vol% THF) solution heat treated (50 °C for 20 min) at every 4 days (a: 1st heat treatment, b: 2nd heat treatment, C: 3rd heat treatment).

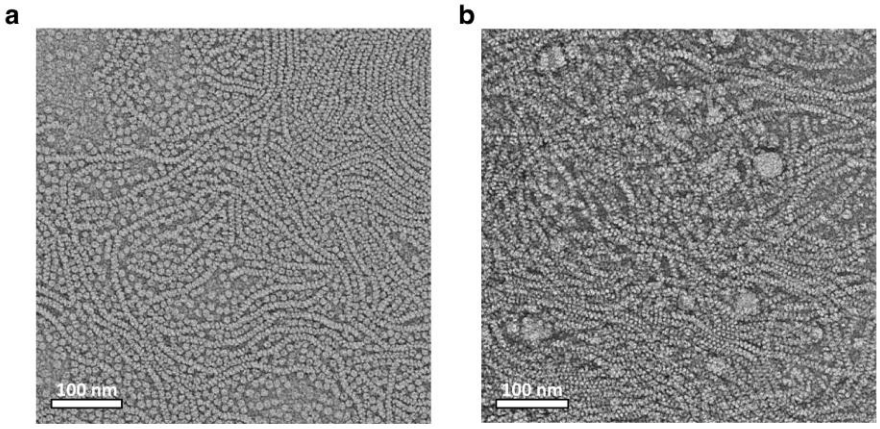

Supplementary Figure 32. Negatively-stained TEM images of helical chains (30 μM of **1*S*** in 10 vol% THF aqueous solution) with subsequent heat treatments at every 4 days before depolymerization undergoes (a: 2nd heat treatment, b: 3rd heat treatment). The results demonstrate that the helical chains can be sustained by continuing energy input.

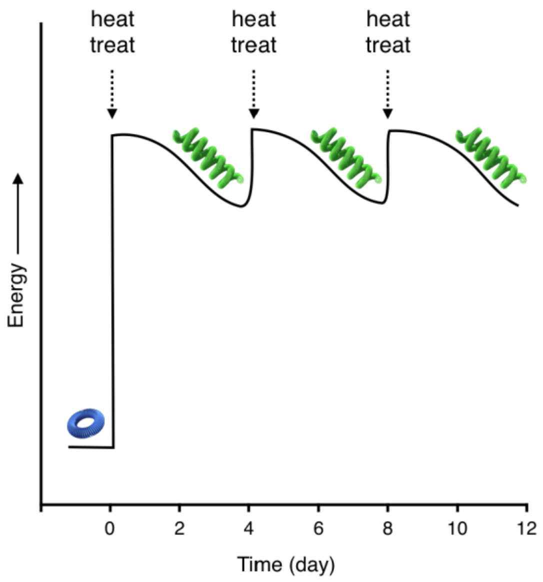

Supplementary Figure 33. The energy landscape of helical chains sustained out-of-equilibrium state by heat-treated (50 ℃ for 20 min) every 4 days.

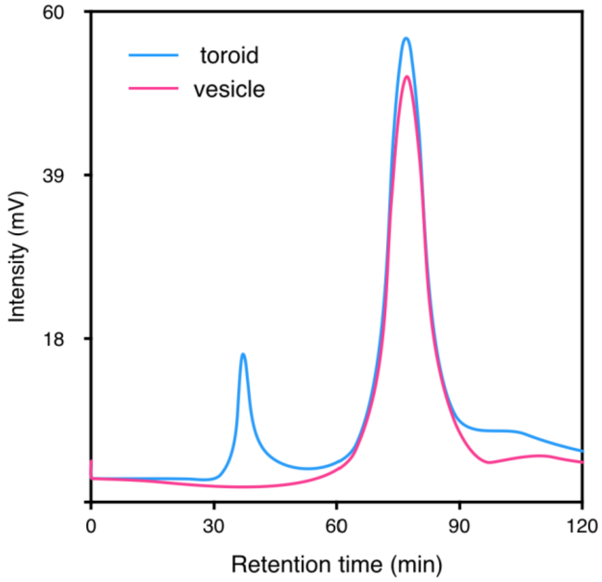

Supplementary Figure 34. Sephacryl chromatograph of toroids solution (blue) and vesicle solution encapsulating toroids (red). The first fraction of vesicle solution was toroids encapsulated by lipid vesicles, and the second fraction was free toroids. The first fraction (30-45 min) was collected to remove non-encapsulated toroids.

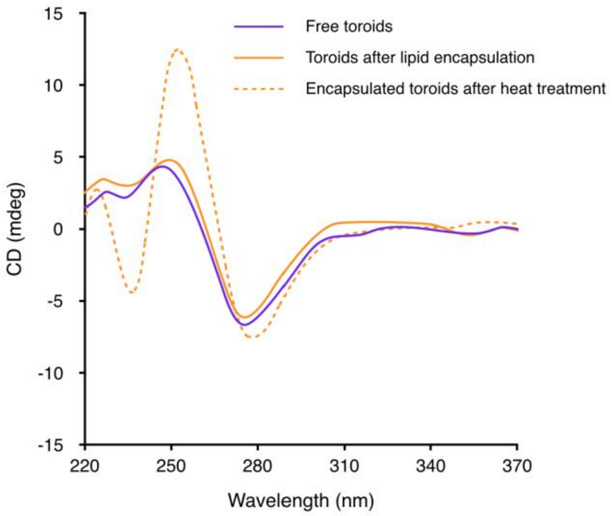

Supplementary Figure 35. CD spectra of **1*S*** (30 μM) in aqueous (10 vol% THF) solution (purple solid line); the vesicle solution by extrusion the mixture of 200 μl DOPC lipid (5 mg/ml) and 200 μl toroids (60 μM) before (orange solid line) and after (orange dash line) heat treatment. The results suggest lipid encapsulation do not disturb toroidal assembly and their helical growth.

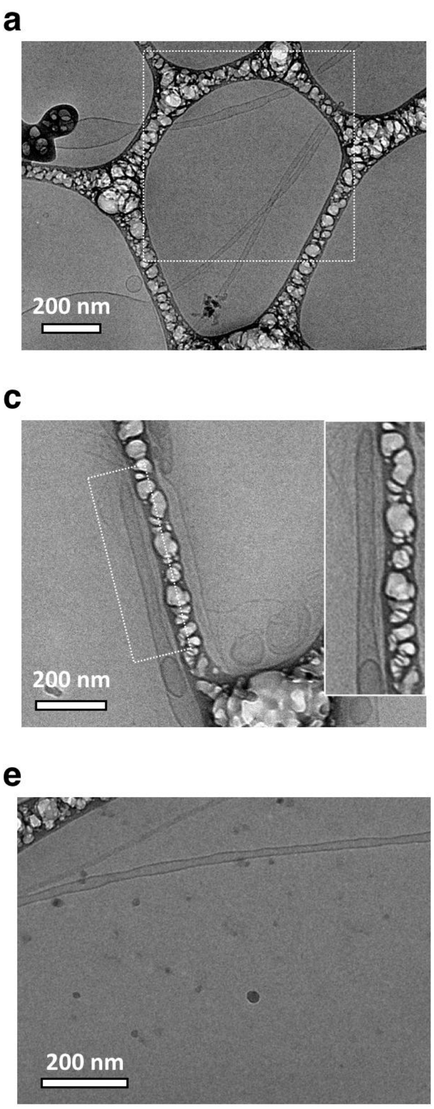

Supplementary Figure 36. Cryo-TEM images of tubular vesicles from vesicle solution which encapsulated toroids after heat treatment (50 ℃ for 20 min) and then standing at room temperature. (a) Original cryo-TEM image corresponding to the area indicated by the white dotted square is shown in Figure 4f. (b)-(f) Cryo-TEM image of another area exhibiting a tubular vesicle.

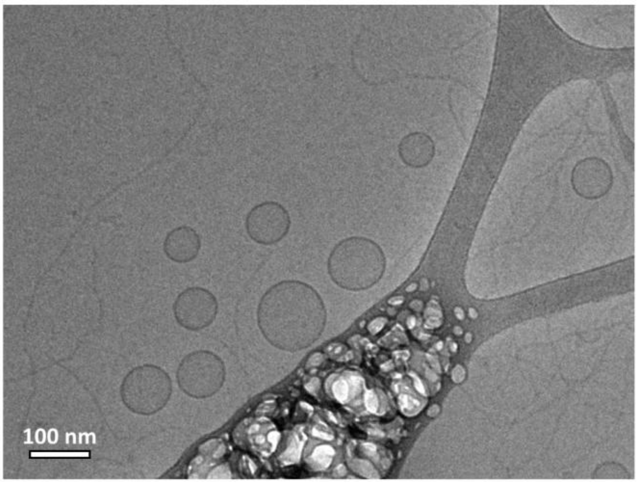

Supplementary Figure 37. Cryo-TEM image of controlled experiments with the mixture solution of toroids of **1** (300 μM) and DOPC (5mg/ml) vesicles without encapsulation of toroids in aqueous (10 vol% THF) solution, after 4 days standing at room temperature of the heat-treated mixture solution. No tubular vesicles were observed, indicating that the shape deformation of vesicles originates from the helical growth of toroids inside vesicles.

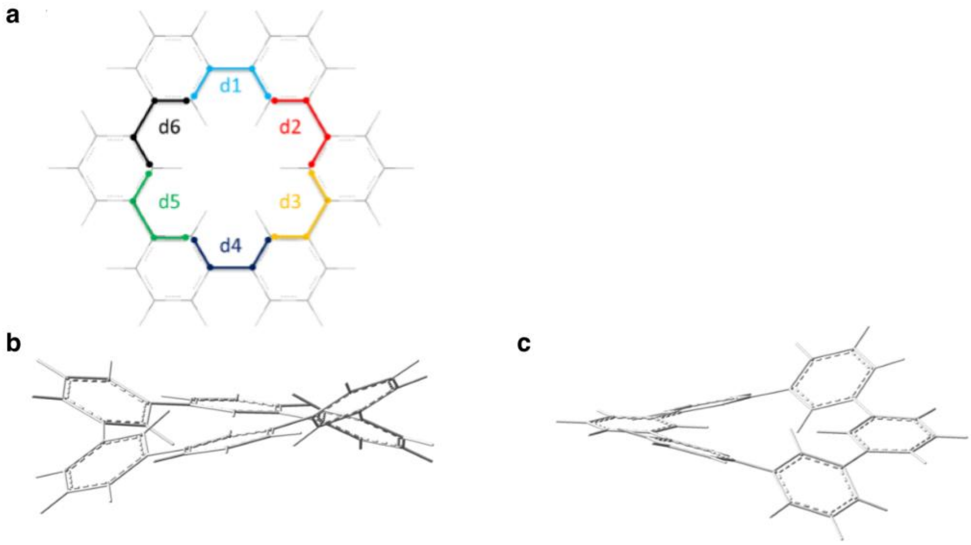

Supplementary Figure 38. The structure of aromatic ring was optimized by quantum chemical calculation using M06l with 6-31g(d,p) basis set implanted in Gaussin09. Based on the optimized ground state structure, the excited state structure was optimized by TDDFT method using PBE1PBE with 6-311g(d) basis set. The conformations of the aromatic structure with (a) representative dihedral angles, (b) before UV irradiation, and (c) after UV irradiation. The specific dihedral angles before and after UV irradiation are listed in the Supplementary Table 1. The results show that UV irradiation drives the aromatic plane to be flatter, resulting in fast depolymerization.

Supplementary Table 1. The angle degree of the dihedral angles.

Dihedrals	Before irradiation (degree)	After irradiation (degree)
d1	-23.45	-13.60
d2	43.89	36.78
d3	-23.40	-23.66
d4	-23.41	-23.66
d5	43.85	36.78
d6	-23.40	-13.59

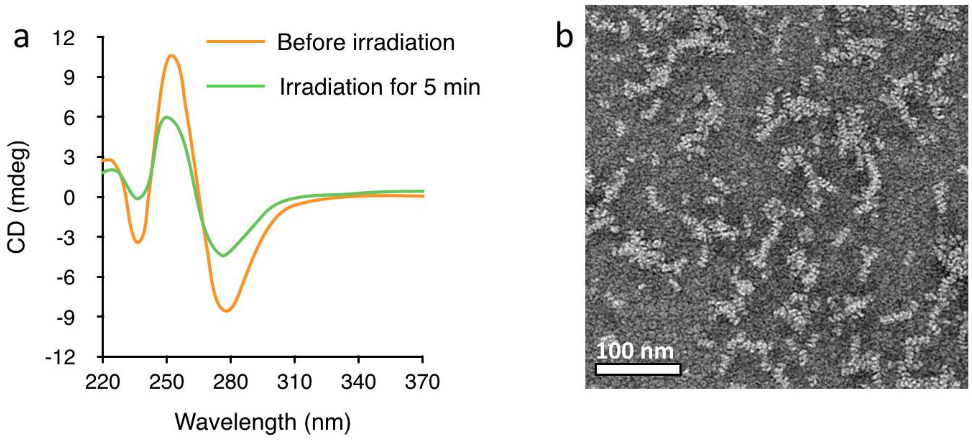

Supplementary Figure 39. (a) CD spectra of **1*S*** (30 μM) in aqueous (10 vol% THF) solution after heat treatment without UV irradiation (orange) and with UV irradiation (254 nm) for 5 min (green). The decrease of CD demonstrates the dimeric macrocycles restored eclipsed conformation by UV irradiation. (b) Negatively-stained TEM image of collapsed polymer chains by UV irradiation (254 nm, 5 min) of a polymer solution obtained from 4 days standing of **1*S*** (30 μM) in aqueous (10 vol% THF) solution after heat treatment (50 °C for 20 min). All of the polymer chains show to be collapsed into short helices and toroids.

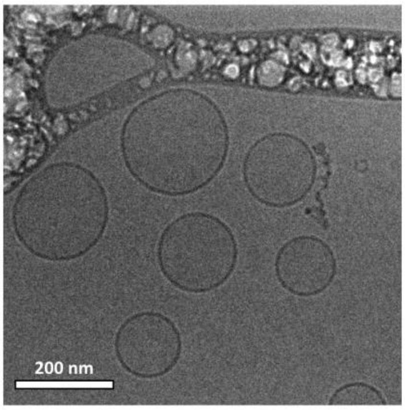

Supplementary Figure 40. Cryo-TEM image of the restored vesicles after UV irradiation (254 nm, 5 min) of tubular vesicles in aqueous (10 vol% THF) solution. Depolymerization of helical chains by UV irradiation drives deformed vesicles to restore spherical shape.

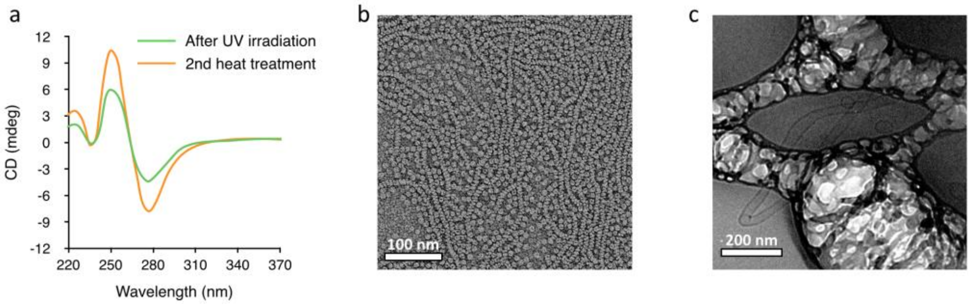

Supplementary Figure 41. (a) CD spectra of the polymer solution **1*S*** (30 μM) in aqueous (10 vol% THF) solution by UV irradiation 5 min (green) and after 2nd heat treatment (orange), indicating the repeatable polymerization. (b) Negatively-stained TEM image of polymer chains by heat treated a collapsed polymer solution obtained from UV irradiation and standing 4 days. (c) Cryo-TEM image of the tubular vesicle from the restored vesicles by UV irradiation after heat treatment and then standing at room temperature.

Supplementary Methods

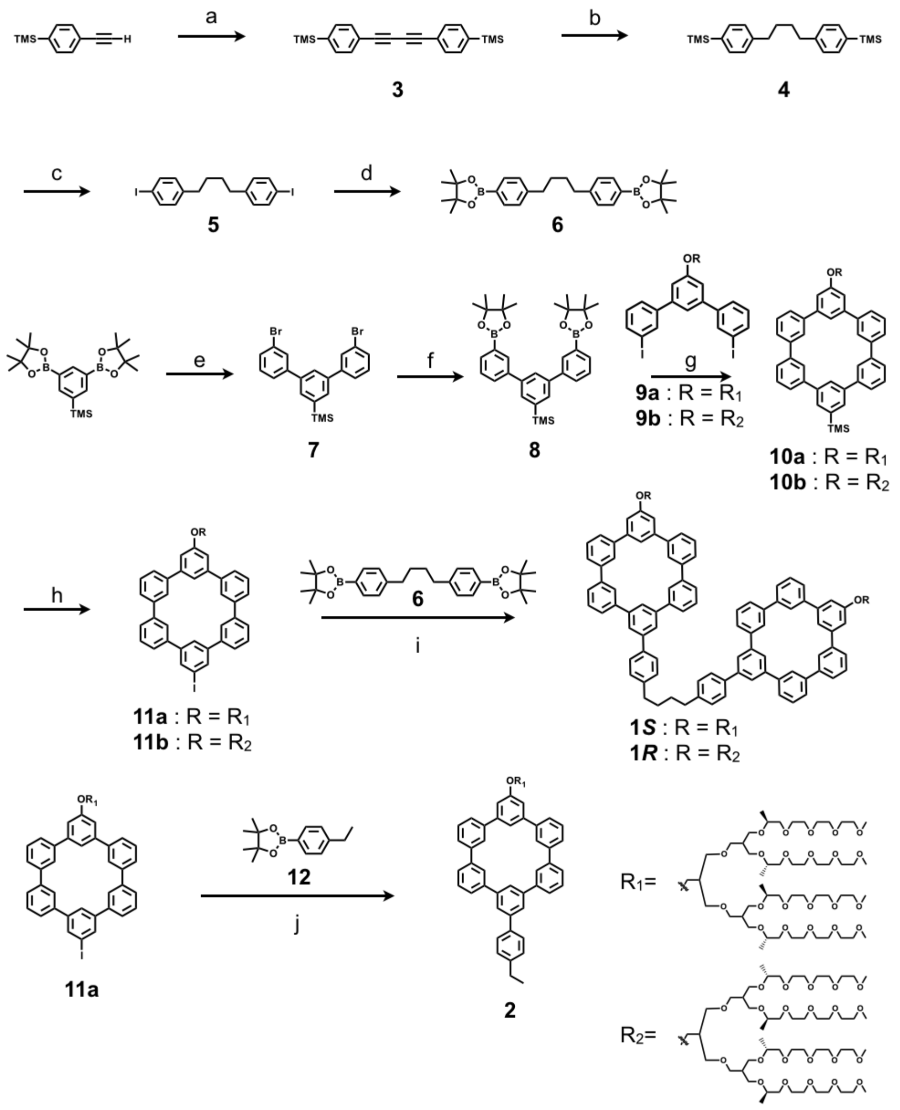

Scheme 1. Synthesis of molecules **1** and **2**.

Reagents and conditions: (a) DBU, TMEDA, CuCl, CH_3CN, rt, 12 h, yield: 50%; (b) Pd/C (Pd 10% on carbon, wetted with ca. 55% water), H_2, MeOH, DCM, rt, yield: 99%; (c) ICl, DCM, 0 ℃, 2 h, yield: 90%; (d) Bis(pinacolato)diboron, Pd(dppf)Cl_2, KOAc, DMSO, 90 ℃, 12 h, yield: 30%; (e) 1-Bromo-3-iodobenzene, Pd(PPh_3)$_4$, Toluene, EtOH, 2 M Na_2CO_3 (*aq*), reflux, 12 h, yield: 50%; (f) Bis(pinacolato)diboron, Pd(dppf)Cl_2, KOAc, DMSO, 90 ℃, 12 h, yield: 75%; (g) Pd(PPh_3)$_4$, Toluene, EtOH, 2 M Na_2CO_3, 80 ℃, 12 h, yield: 30%; (h) ICl, DCM, 0 ℃, 2 h, yield: 38%; (i) Pd(PPh_3)$_4$, Toluene, EtOH, 2 M Na_2CO_3, 80 ℃, 12 h, yield: 60%; (j) Pd(PPh_3)$_4$, Toluene, EtOH, 2 M Na_2CO_3, 80 ℃, 12 h, yield: 50%.

Compound 3. DBU (104 mg, 0.69 mmol), TMEDA (1.1 mg, 0.001 mmol), and CuCl (1.4 mg, 0.014 mmol) were added at room temperature under stirring to 0.7 ml of acetonitrile while oxygen was bubbled through the solution. After 10 min, 2, 4, 5-trimethyl phenylacetylene (120 mg, 0.7 mmol) was added. The reaction mixture was stirred at room temperature overnight. After completion of the reaction as monitored by TLC, the solvent was removed under reduced pressure and extraction by DCM and brine. The organic solution dried over $MgSO_4$ (s). The organic phase was filtered with Celite and the filtrate was condensed in a rotary evaporator. The crude product was purified by flash silica gel column chromatography (eluent condition: hexane) to provide 50% yield of a white solid.

^{1}H-NMR (500 MHz, Chloroform-*d*) δ 7.49 (s, 8H), 0.27 (s, 18H).

Compound 4. In a typical reaction compound **3** (50 mg, 0.14 mmol) was dissolved in methanol and dichloromethane (1:1, v/v) 1ml. 10 mg of Pd/C was added in the solution and was stirred under H_2 for 2 hours. After completion of the reaction as monitored by TLC, the reaction mixture was filtered by Celite. The product provides 99% yield of a white solid without further purifications.

^{1}H-NMR (500 MHz, Chloroform-*d*) δ 7.43 (d, J = 7.6 Hz, 4H), 7.17 (d, J = 7.5 Hz, 4H), 2.63 (t, J = 5.8 Hz, 4H), 1.68 (t, J = 6.8 Hz, 4H), 0.25 (s, 18H).

Compound 5. Compound **4** (50 mg, 0.14 mmol) was dissolved in anhydrous dichloromethane (DCM) (1.5 ml). ICl (1.0 M in DCM solution) (0.32 ml, 0.32 mmol) was added dropwise to the reactant solution at 0 ℃ ice bath. The mixture was stirred in 0 ℃ for 2 hours under argon. After completion of the reaction as monitored by TLC, the reaction was quenched with $Na_2S_2O_3$ saturated aqueous solution. The organic layer was washed with brine and dried over $MgSO_4$ (s). The organic phase was filtered with Celite and the filtrate was condensed in a rotary evaporator. The crude product was purified by flash silica gel column chromatography (eluent condition: hexane) to provide 90% yield.

^{1}H-NMR (500 MHz, Chloroform-*d*) δ 7.58 (d, J = 8.3 Hz, 4H), 6.90 (d, J = 8.1 Hz, 4H), 2.56 (t, J = 6.3 Hz, 4H), 1.60 (t, J = 3.3 Hz, 4H).

Compound 6. Compound **5** (50 mg, 0.11 mmol) and bis(pinacolato)diboron (60 mg, 0.24 mmol) were dissolved in degassed DMSO (0.5 ml), then Pd(dppf)Cl_2 (49 mg, 0.006 mmol) and KOAc (63 mg, 0.6 mmol) were added carefully. The mixture was heated to 90 ℃ for overnight. After completion of the reaction as monitored by TLC, the reaction mixture was cooled to room temperature and was extracted with ethyl acetate. The combined organic layers were dried over anhydrous $MgSO_4$ (s). The organic phase was filtered with Celite and the filtrate was condensed in a rotary evaporator. The crude product was purified by silica gel flash column chromatography (eluent condition: ethyl acetate/hexane = 1/20) to provide 30% yield of a white solid.

^{1}H-NMR (500 MHz, Chloroform-*d*) δ 7.71 (d, *J* = 7.9 Hz, 4H), 7.17 (d, *J* = 7.8 Hz, 4H), 2.63 (t, *J* = 6.5 Hz, 4H), 1.65 (p, *J* = 3.9 Hz, 4H), 1.33 (s, 24H).

Compound 7. 3,5-Dibromo-1-trimethylsilylbenzene (1.5 g, 3.76 mmol) and 1-bromo-3-iodobenzene (2.1 g, 7.5 mmol) were dissolved in degassed toluene (37 ml), EtOH (18 ml) and 2 M aqueous Na_2CO_3 (9 ml), then Pd(PPh_3)$_4$ (43 mg, 0.04 mmol) was added to the mixture. The mixture was refluxed overnight under argon. After completion of the reaction as monitored by TLC, the reaction mixture was cooled to room temperature and was extracted with ethyl acetate. The combined organic layers were dried over anhydrous $MgSO_4$ (s). The organic phase was filtered with Celite and the filtrate was condensed in a rotary evaporator. The crude product was purified by flash silica gel column chromatography (eluent condition: hexane) to provide 50% yield.

^{1}H-NMR (500 MHz, Chloroform-*d*) δ 7.76 (t, *J* = 1.9 Hz, 2H), 7.66 (s, 3H), 7.55 (dt, *J* = 7.7, 1.4 Hz, 2H), 7.51 (ddd, *J* = 8.0, 2.0, 1.0 Hz, 2H), 7.34 (t, *J* = 7.8 Hz, 2H), 0.35 (s, 9H).

Compound 8 was prepared by the synthetic method of compound **6**. The crude product was purified by silica gel flash column chromatography (eluent condition: ethyl acetate/hexane = 1/15) to provide 75% yield of a white solid.

^{1}H-NMR (500 MHz, Chloroform-*d*) δ 8.05 (s, 2H), 7.82 (dt, *J* = 7.3, 1.2 Hz, 2H), 7.79 (t, *J* = 1.9 Hz, 1H), 7.73 (dt, *J* = 7.8, 1.6 Hz, 2H), 7.68 (d, *J* = 1.9 Hz, 2H), 7.47 (t, *J* = 7.6 Hz, 2H),

1.37 (s, 24H), 0.35 (s, 9H).

Compound 10a and 10b. Compound **9a** (600 mg, 0.38 mmol), compound **8** (210 mg, 0.38 mmol) were dissolved in distilled toluene (114 ml), EtOH (57 ml) and 2 M aqueous Na_2CO_3 (30 ml). The mixture was purged with argon for 5 min. Then $Pd(PPh_3)_4$ (1.3 mg, 0.004 mmol) was added carefully. The mixture was stirred at 80 ℃ overnight under argon. After completion of the reaction as monitored by TLC, the reaction mixture was cooled down to room temperature and was diluted with ethyl acetate and washed with brine. The combined organic phase was dried over anhydrous $MgSO_4$ (s). The filtrate was condensed under reduced pressure and purified by silica gel flash column (eluent condition: methanol/ethyl acetate = 1/20) to provide 30% yield of a yellow waxy solid.

^{1}H-NMR (500 MHz, Chloroform-*d*) δ 8.35 (s, 2H), 8.32 (s, 2H), 8.31 (s, 1H), 7.93 (s, 1H), 7.86 (d, *J* = 1.7 Hz, 2H), 7.74 (m, 8H), 7.58 (q, *J* = 7.8 Hz, 4H), 7.28 (d, *J* = 1.4 Hz, 2H), 4.20 (d, *J* = 5.6 Hz, 2H), 3.70 – 3.30 (m, 88H), 2.45 (p, *J* = 5.9 Hz, 1H), 2.11 – 2.08 (m, 2H), 1.09 (d, *J* = 6.2 Hz, 12H), 0.39 (s, 9H). MALDI-TOF mass: *m/z* calcd. for $C_{91}H_{136}NaO_{23}Si$ $[M + Na]^+$, 1647.91; found, 1647.27.

Compound 11a and 11b were prepared by the synthetic method of compound **5**. The crude product was purified by silica gel flash column chromatography (eluent condition: methanol/ethyl acetate = 1/20) and further purification by prep-HPLC (C8 column, ACN/H_2O = 90/10) to provide 38% yield of a white solid.

^{1}H-NMR (500 MHz, Chloroform-*d*) δ 8.31 (s, 2H), 8.27 (s, 3H), 8.06 (d, *J* = 1.4 Hz, 2H), 7.91 (s, 1H), 7.76 – 7.71 (m, 6H), 7.68 (d, *J* = 8.0 Hz, 2H), 7.57 (t, *J* = 7.7 Hz, 4H), 7.27 (d, *J* = 1.5 Hz, 2H), 4.20 (d, *J* = 5.7 Hz, 2H), 3.63 – 3.30 (m, 88H), 2.45 (p, *J* = 5.9 Hz, 1H), 2.10 (p, *J* = 5.8 Hz, 2H), 1.09 (d, *J* = 6.2 Hz, 12H). MALDI-TOF mass: *m/z* calcd. for $C_{88}H_{127}INaO_{23}$ $[M + Na]^+$, 1701.77; found, 1701.48.

Compound 1*S* and 1*R*. These compounds were synthesized using the same procedure. A representative example is described by **1*S***. Compound **11a** (77 mg, 0.05 mmol), compound **6**

(11 mg, 0.02 mmol) were dissolved in distilled toluene (1 ml), EtOH (0.5 ml) and 2M aqueous Na_2CO_3 (0.4 ml). The mixture was purged with argon for 5 min. Then $Pd(PPh_3)_4$ (0.8 mg, 0.001 mmol) was added carefully. The mixture was stirred at 80 ℃ overnight under argon. After completion of the reaction as monitored by TLC, the reaction mixture was cooled down to room temperature and was diluted with ethyl acetate and washed with brine. The combined organic phase was dried over anhydrous $MgSO_4$ (s). The filtrate was condensed under reduced pressure and purified by silica gel flash column (eluent condition: methanol/ethyl acetate = 1/3) and further purification by prep-HPLC (C8 column, ACN 100%) to provide 60% yield of a white waxy solid.

^{1}H-NMR (500 MHz, Chloroform-*d*) δ 8.38 (d, *J* = 7.7 Hz, 8H), 8.32 (s, 2H), 7.96 (s, 2H), 7.93 (s, 4H), 7.80 (d, *J* = 7.7 Hz, 4H), 7.74 (t, *J* = 8.8 Hz, 12H), 7.68 (d, *J* = 7.7 Hz, 4H), 7.59 (q, *J* = 7.0 Hz, 8H), 7.35 (d, *J* = 7.7 Hz, 4H), 7.28 (s, 4H), 4.20 (d, *J* = 5.6 Hz, 4H), 3.67 – 3.30 (m, 176H), 2.78 (t, *J* = 5.5 Hz, 4H), 2.48 – 2.42 (m, 2H), 2.10 (p, *J* = 6.1 Hz, 4H), 1.81 (t, *J* = 5.5 Hz, 4H), 1.09 (d, *J* = 6.2 Hz, 24H). ^{13}C-NMR (126 MHz, Chloroform-*d*) δ 160.49, 142.82, 142.56, 142.28, 141.82, 141.37, 141.27, 129.65, 129.15, 127.51, 127.23, 127.15, 125.81, 124.61, 111.93, 77.41, 77.16, 76.91, 75.20, 74.98, 72.10, 70.91, 70.76, 70.68, 69.83, 69.57, 67.60, 66.57, 59.17, 41.03, 40.33, 35.70, 31.29, 29.86, 17.30, 0.15. MALDI-TOF mass: *m/z* calcd. for $C_{192}H_{270}NaO_{46}$ $[M + Na]^+$, 3334.87; found, 3335.13.

Compound 2 was prepared by the synthetic method of compound **1**. The crude product was purified by silica gel flash column chromatography (eluent condition: methanol/ethyl acetate = 1/20) and further purification by prep-HPLC (C8 column, ACN/ H_2O = 90/10) to provide 50% yield of a white waxy solid.

^{1}H-NMR (500 MHz, Chloroform-*d*) δ 8.38 (d, *J* = 6.4 Hz, 4H), 8.32 (s, 1H), 7.96 (s, 1H), 7.93 (d, *J* = 1.6 Hz, 2H), 7.80 (d, *J* = 7.6 Hz, 2H), 7.74 (tt, *J* = 8.3, 6.8, 2.8 Hz, 6H), 7.68 (d, *J* = 8.1 Hz, 2H), 7.59 (q, *J* = 7.9 Hz, 4H), 7.36 (d, *J* = 8.0 Hz, 2H), 7.28 (d, *J* = 1.5 Hz, 2H), 4.20 (d, *J* = 5.6 Hz, 2H), 3.65 – 3.31 (m, 88H), 2.75 (q, *J* = 7.6 Hz, 2H), 2.45 (p, *J* = 5.9 Hz, 1H), 2.10 (p, *J* = 5.9 Hz, 2H), 1.32 (t, *J* = 7.6 Hz, 3H), 1.09 (d, *J* = 6.1 Hz, 12H). ^{13}C-NMR (126 MHz, Chloroform-*d*) δ 160.44, 143.98, 142.83, 142.53, 141.79, 141.37, 141.33, 141.24, 138.75,

129.65, 129.62, 128.56, 127.51, 127.22, 127.13, 125.82, 125.77, 125.72, 125.68, 124.61, 119.56, 111.88, 77.41, 77.16, 76.91, 75.15, 74.96, 74.94, 72.07, 70.88, 70.75, 70.73, 70.65, 69.78, 69.76, 69.53, 67.56, 66.50, 59.16, 40.98, 40.27, 28.73, 27.06, 17.27, 15.78, 0.15. MALDI-TOF mass: *m/z* calcd. for $C_{96}H_{136}NaO_{23}$ $[M + Na]^+$, 1679.94; found, 1680.38.

All-atom simulations

Molecular structure was first optimized by quantum chemical calculation using M062x with 6-31g(d,p) basis set implanted in Gaussin09. Then the partial charges of atoms in the molecule were determined by R.E.D. (RESP ESP charge Derive) method, and other parameters were assigned according to general AMBER force field[1]. The TIP3P water model was chosen in the simulations. The all-atom molecular dynamics (AAMD) simulations were performed using NAMD package[2] in isothermal-isobaric (NPT) ensemble. The temperature was set as 298 K and the pressure was set as 1 atm. The cutoff radius of van der Waals interaction was set as 12 Å and the integration time step was 2 fs. Particle mesh Ewald method was used for the calculation of electrostatic interaction with grid spacing of 1.0 Å.

Coarse-grained model and simulations

As illustrated in Supplementray Figure 42, we built a coarse-grained (CG) model for the molecular self-assembly building block, in which a group of atoms with similar size were represented by a CG particle.

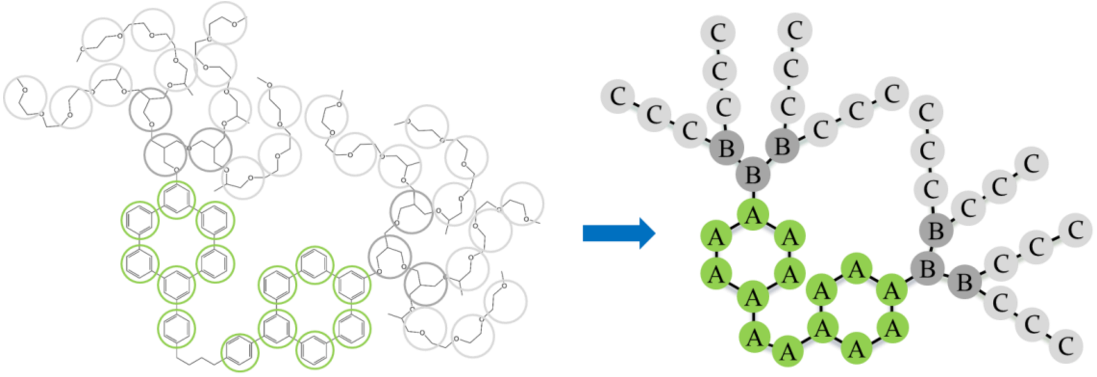

Supplementary Figure 42. Illustration of CG scheme.

To be specific, the phenyl group in dimeric aromatic rings is represented by CG particle with type A. The oligoether chain segment with five heavy atoms including carbon and oxygen is represented by particle with type B or C, depending on the detailed chemical composition. The type B particle represents chain segment that has more carbon atoms and is more hydrophobic than particle with type C. Similar to MARTINI force field[3], four water molecules are represented by a CG particle assigned with type W. The CG scheme is illustrated in Supplementray Figure 42.

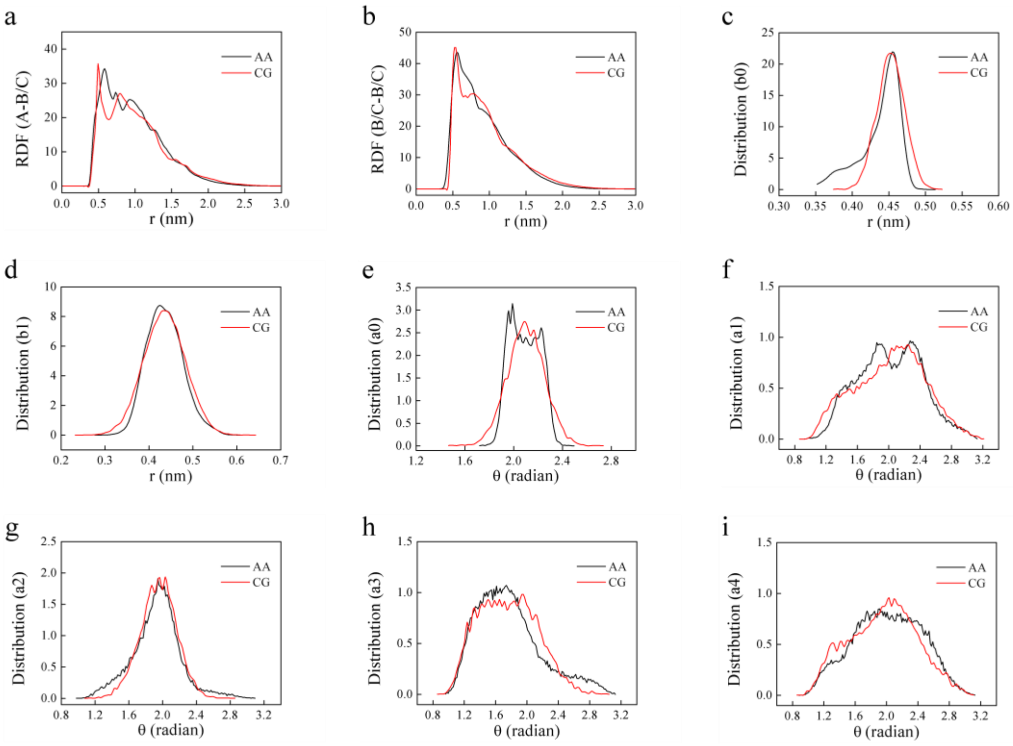

Supplementary Figure 43. Structural fitting of CG model to AA model for non-bonded (a), (b), bond (c), (d), and angle (e), (f), (g), (h), (i) potentials.

We derived CG force field by a hybrid coarse-graining method, in which the key structures of CG model were fitted to the ones obtained from AAMD simulations. In both the AAMD and the CGMD simulations, we considered the system with one molecular self-assembly building block and 3296 water molecules at the same thermodynamic condition. The interaction parameters of CG model were continually adjusted until the key structures obtained in CG model can be best-fitted to the ones obtained in AAMD simulations. The best-fitted structural

distributions from AAMD and CGMD simulations are shown in Supplementary Figure 43. All CGMD simulations in this work were performed using GALAMOST package[4,5].

We employed Lennard-Jones potential (1) for non-bonded interactions between CG particles:

$$V_{LJ}(r) = 4\varepsilon\left[\left(\frac{\sigma}{r}\right)^{12} - \left(\frac{\sigma}{r}\right)^{6}\right] \quad (1)$$

Supplementary Table 2 The parameters of non-bonded interactions

	**A**	**B**	**C**	**W**
**A**	IV	IV	IV	V
**B**		IV	IV	III
**C**			IV	II
**W**				I
I, ε = 5.0 kJ/mol; II, ε = 4.0 kJ/mol; III, ε = 3.6 kJ/mol; IV, ε = 3.0 kJ/mol; V, ε = 2.3 kJ/mol; σ_{AA}=0.35 nm; $\sigma_{BB} = \sigma_{CC} = \sigma_{WW}$ = 0.47 nm.				

The non-bonded interaction parameters were $\varepsilon_{W\text{-}W}$ = 5.0 kJ/mol, $\varepsilon_{A\text{-}A}$ = 3.0 kJ/mol, $\varepsilon_{A\text{-}B(C)}$ = 3.0 kJ/mol, $\varepsilon_{B(C)\text{-}B(C)}$ = 3.0 kJ/mol, and $\varepsilon_{A\text{-}W}$ = 2.3 kJ/mol. The key interaction parameters $\varepsilon_{B\text{-}W}$ and $\varepsilon_{C\text{-}W}$ that control the self-assembly behavior were derived from the solvation energies of oligoether chain segments. The B and C particles represent the chain segments of 5/3 poly(ethylene oxide) (PEO) and 5/4 poly(propylene oxide) (PPO) units, respectively. Their respective solvation energies $\Delta E_B = \varepsilon_{B\text{-}W} - (\varepsilon_{B\text{-}B} + \varepsilon_{W\text{-}W})/2$ and $\Delta E_C = \varepsilon_{C\text{-}W} - (\varepsilon_{C\text{-}C} + \varepsilon_{W\text{-}W})/2$ could be compared to the reported values ΔE_{PEO} = -0.0625 kJ/mol and ΔE_{PPO} = 0.3125 kJ/mol[6]. The obtained parameters $\varepsilon_{B\text{-}W}$ = 3.6 kJ/mol and $\varepsilon_{C\text{-}W}$ = 4.0 kJ/mol were confirmed reasonably by comparing the radial distribution functions between A and B(C) and between B(C) and B(C) with the ones of AAMD simulations (Supplementary Figure 43a, b). The particle size parameter σ between two different types of particles could be calculated by $\sigma_{\alpha\beta} = (\sigma_\alpha + \sigma_\beta)/2$ with α, β = A, B, C, or W. All non-bonded interaction parameters are listed in Supplementary Table 2.

The harmonic potential was used to describe bond-stretching (2). The bond between A and B particles is set as type b0, and the bonds between similar types of particles such as B-B, B-C, and C-C are regarded as the same type b1. Equilibrium lengths r_0 and force constants K_{bond}

were obtained by fitting to the bond length distributions derived from AAMD simulations, as listed in Supplementary Table 3.

$$V_{bond}(r) = \frac{1}{2}K_{bond}(r - r_0)^2 \quad (2)$$

Supplementary Table 3 The parameters of bond-stretching potentials

**Bonds**	**Types**	r_0 **(nm)**	K_{bond} **(kJ mol^{-1} nm^{-2})**
A-B	b0	0.46	3750
B-B	b1	0.46	1250
B-C	b1	0.46	1250
C-C	b1	0.46	1250

The harmonic form of potential was also used to describe the bond angle-bending in CG models (3). Different types of bond angles are listed in Supplementay Table 4. Equilibrium angles Θ_0 and force constants K_{angle} were obtained by fitting to the bond angle distributions derived from AAMD simulations, as listed in Supplementary Table 4..

$$V_{angle}(r) = \frac{1}{2}K_{angle}(\theta - \theta_0)^2 \quad (3)$$

Supplementary Table 4 The parameters of bond angle-bending potentials

**Angles**	**Types**	Θ_0 **(degree)**	K_{angle} **(kJ mol^{-1})**
A-A-B	a0	140.0	50.0
A-B-B	a1	135.0	10.0
B-B-B	a2	115.0	50.0
B-B-C	a1	135.0	10.0
C-B-C	a2	115.0	50.0
B-C-C	a3	100.0	14.0
C-C-C	a4	120.0	8.0

To reduce the complexity of our CG model, we used rigid-body method[5,7] to control the whole aromatic dimer at a fixed configuration and thereby a fixed slip distance. The configuration of aromatic dimer was taken from the one optimized by AAMD simulations.

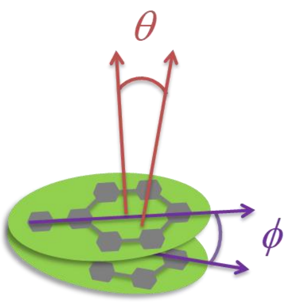

Supplementary Figure 44. Illustration of π-π interaction model.

The face-to-face (i.e., the π-π) interaction between aromatic rings is much stronger than the edge-to-edge interaction. However, typically in CG models, the strength of face-to-face interaction is underestimated and normally coupled to edge-to-edge interaction. To reflect correct anisotropic interactions between aromatic rings, we introduced an additional face-to-face potential as illustrated in Supplementary Figure 44[8] with the form given by (4):

$$V_{\pi-\pi}(r) = -\varepsilon cos^2(\theta)cos^2(3\phi)(r_{cut-off} - r)^2 \quad (4)$$

where r is the distance between aromatic ring centers, and as shown in Supplementay Figure 44, θ and ϕ are the angles between normal vectors and axial vectors of two aromatic planes, respectively. The parameters ε = 350 kJ mol^{-1} nm^{-2} and $r_{cut\text{-}off}$ = 0.66 nm ensure the minimum energy $V_{\pi\text{-}\pi}$ = -42.8 kJ/mol with r = 0.31 nm and the total minimum energy $V_{\pi\text{-}\pi}$ + V_{LJ} = -63.8 kJ/mol[9].

The kinetic Monte Carlo simulations of polymerization/depolymerization of toroids.

We performed kinetic Monte Carlo (KMC) simulations to reveal the mechanism of polymerization and depolymerization based on available experimental data[10]. In the KMC simulations, the basic unit that we focus on is the molecular self-assembly building block, while the polymerization unit is the toroid that consists of 27 molecules. The packing mode of aromatic dimers is initially slipped in simulations, in correspondence to the system after heating. When the temperature goes back to the room temperature, the slipped state is energetically unstable as compared to the eclipsed one, therefore the slipped state will slowly decay to the

eclipsed state with a first order kinetics. It means the decay rate is only proportional to the concentration of slipped molecules.

Supplementary Table 5 The parameters of kinetic Monte Carlo simulations

Parameters	Value
$\boldsymbol{N}$	1×10^4
$\boldsymbol{P_e}$	2.0×10^{-4} /(10 minutes)
$\boldsymbol{S_p}$	60%
$\boldsymbol{P_r}$	0.6/N/(10 minutes)
$\boldsymbol{K}$	5.5×10^{-3}
$\boldsymbol{P_d}$	1.0×10^{-3} /(10 minutes)

The simulation is discrete in the time unit of 10 minutes. In the simulation, each slipped molecule has a probability over a period (P_e) to become eclipsed. Initially, N toroids with all the constituent molecules in slipped state are active for polymerization. When the proportion of slipped molecule in a toroid is lower than S_p, which is estimated from energy profile, the toroid becomes inactive.

$$P = \begin{cases} P_r K^2 & for\ N_i = 1\ and\ N_j = 1 \\ P_r\left(\frac{1}{N_i^3} + \frac{1}{N_j^3}\right) & for\ others \end{cases} \qquad (5)$$

The initial reaction probability over a period of time by two active toroids (P_rK^2) is small due to that they have to transform from the closed state to open state with an equilibrium constant K between open toroid and closed toroid states.

Considering the diffusion and rotation of linear polymer, the polymerization probability should be inversely proportional to the third power of polymer length that is described by the number of toroids in a polymerized structure (N_i and N_j in (5)). Thereby, we employ P in (5) to describe the possibility of polymerization between two objects, i. e. active toroids and polymers. The polymerization probability factor P_r is related to the total toroid number N to eliminate system size effect.

For each pair of neighboring inactive toroids in a polymerized structure, there is a probability over a period (P_d) to break the connection and the depolymerization takes place.

Supplementary References

[1]. Wang, J., Wolf, R. M., Caldwell, J. W., Kollman, P. A. & Case, D. A. Development and testing of a general amber force field. *J. Comput. Chem.* **25,** 1157–1174 (2004).

[2]. Phillips, J. C. et al. Scalable molecular dynamics with NAMD. *J. Comput. Chem.* **26,** 1781–1802 (2005).

[3]. de Jong, D. H. et al. Improved parameters for the martini coarse-grained protein force field. *J. Chem. Theory Comput.* **9,** 687–697 (2013).

[4]. Zhu, Y.-L. et al. GALAMOST: GPU-accelerated large-scale molecular simulation toolkit. *J. Comput. Chem.* **34,** 2197-2211 (2013).

[5]. Zhu, Y.-L. et al. Employing multi-GPU power for molecular dynamics simulation: an extension of GALAMOST. *Mol. Phys.* **116,** 1065–1077 (2018).

[6]. Nawaz, S. & Carbone, P. Coarse-graining poly(ethylene oxide)–poly(propylene oxide)–poly(ethylene oxide) (PEO–PPO–PEO) block copolymers using the MARTINI force field. *J. Phys. Chem. B* **118,** 1648–1659 (2014).

[7]. Nguyen, T. D., Phillips, C. L., Anderson, J. A. & Glotzer, S. C. Rigid body constraints realized in massively-parallel molecular dynamics on graphics processing units. *Comput. Phys. Commun.* **182,** 2307–2313 (2011).

[8]. Lin, Y.-L., Chang, H.-Y., Sheng, Y.-J. & Tsao, H.-K. Photoresponsive polymersomes formed by amphiphilic linear–dendritic block copolymers: generation-dependent aggregation behavior. *Macromolecules* **45,** 7143–7156 (2012).

[9]. Hayashi, T. & Kinoshita, M. Statistical thermodynamics of aromatic–aromatic interactions in aqueous solution. *Phys. Chem. Chem. Phys.* **18,** 32406–32417 (2016).

[10]. Shi, X.-q. & Ma, Y.-q. Understanding phase behavior of plant cell cortex microtubule organization. *PNAS* **107,** 11709–11714 (2010).
