# Supplementary material for: Autonomous helical propagation of active toroids with mechanical action
Source: Nat Commun. 2019 Mar 6;10:1080. doi: 10.1038/s41467-019-09099-9 (PMC6403424; doi:10.1038/s41467-019-09099-9)
Supplement: Supplementary file 2 — Description of Additional Supplementary Files [file 41467_2019_9099_MOESM2_ESM.pdf]

Description of Additional Supplementary Files

File Name: Supplementary Movie 1
Description: All-atom molecular dynamic simulations of inactive toroid.

File Name: Supplementary Movie 2
Description: Coarse-grained molecular dynamic simulations of active toroid.
